# HrpA, a DEAH-Box RNA Helicase, Is Involved in Global Gene Regulation in the Lyme Disease Spirochete

**DOI:** 10.1371/journal.pone.0022168

**Published:** 2011-07-26

**Authors:** Aydan Salman-Dilgimen, Pierre-Olivier Hardy, Ashley R. Dresser, George Chaconas

**Affiliations:** Departments of Biochemistry and Molecular Biology and Microbiology and Infectious Diseases, University of Calgary, Calgary, Alberta, Canada; Monash University, Australia

## Abstract

Spirochetes causing Lyme borreliosis are obligate parasites that can only be found in a tick vector or a vertebrate host. The ability to survive in these two disparate environments requires up and downregulation of specific genes by regulatory circuits that remain largely obscure. In this work on the Lyme spirochete, *B. burgdorferi*, we show that a disruption of the *hrpA* gene, which encodes a putative RNA helicase, results in a complete loss in the ability of the spirochetes to infect mice by needle inoculation. Studies of protein expression in culture by 2D gels revealed a change in the expression of 33 proteins in *hrpA* clones relative to the wild-type parent. Quantitative characterization of protein expression by iTRAQ analysis revealed a total of 187 differentially regulated proteins in an *hrpA* background: 90 downregulated and 97 upregulated. Forty-two of the 90 downregulated and 65 of the 97 upregulated proteins are not regulated under any conditions by the previously reported regulators in *B. burgdorferi* (*bosR*, *rrp2*, *rpoN*, *rpoS* or *rrp1*). Downregulated and upregulated proteins also fell into distinct functional categories. We conclude that HrpA is part of a new and distinct global regulatory pathway in *B. burgdorferi* gene expression. Because an HrpA orthologue is present in many bacteria, its participation in global regulation in *B. burgdorferi* may have relevance in other bacterial species where its function remains obscure. We believe this to be the first report of a role for an RNA helicase in a global regulatory pathway in bacteria. This finding is particularly timely with the recent growth of the field of RNA regulation of gene expression and the ability of RNA helicases to modulate RNA structure and function.

## Introduction

Lyme borreliosis is common in the northern hemisphere and is now the most frequent tick-borne disease in North America and Europe [1], [2]. The causative agents, *Borrelia burgdorferi* and related species, are obligate parasites that survive through a complex enzootic cycle involving a tick vector and a vertebrate host. Differential gene regulation in these two environments is an important feature for successful adaptation to both the tick vector and the infected animal (see [3], [4], [5] for recent reviews). About 150 genes appear to be differentially regulated in *B. burgdorferi*, depending upon environmental conditions and tick or host factors required for survival in these very different settings [6], [7], [8], [9], [10], [11], [12], [13], [14], [15], [16].

There is much that remains unknown about the regulatory pathways in *Borrelia* species, which as in other organisms can function at the transcriptional and translational stages and at steps in between. Studies in *B. burgdorferi* are complicated by the need for growth in ticks and animals to accurately characterize global gene regulation. Nonetheless, several different regulatory molecules and pathways that play a role in differential gene expression have been identified, including the BosR regulator [5], [14], [15], [16], [17], [18], alternative σ factors RpoS and RpoN [7], [12], [19], [20] and the RpoN activator Rrp2 [21], [22], [23]. The two-component response regulatory system Rrp1-Hpk1 [24] as well as the DNA binding and bending protein Hbb [25], and DNA supercoiling [26], [27] also play a role in the modulation of gene expression in *B. burgdorferi*. RNA regulation has also been recently reported in *B. burgdorferi*; the RNA regulator DsrA has been shown to regulate the expression of *rpoS* and *ospC* [28] and the RNA chaperone Hfq appears to be involved in regulating the expression of pathogenicity factors [29].

RNA helicases are important enzymes present in virtually all living organisms. They unwind double stranded RNA in an energy-dependent manner and are involved in a wide variety of RNA metabolic functions [30], [31], [32], [33]. HrpA, a DEAH-box RNA helicase has been shown to be involved in processing of *daa* mRNA from a fimbrial operon in *E. coli*. The processing event results in a stable mRNA and upregulation of *daa* expression relative to other proteins encoded by the polycistronic transcript [34]. The HrpA protein also appears to be involved in physical interactions with a variety of ribosomal proteins in *E. coli*, either directly, or indirectly through RNA interaction [35], consistent with a possible regulatory role at the translational level. Other than these two reports, no other information on the function of HrpA in any bacterium exists in the current literature.

**Figure 1:**
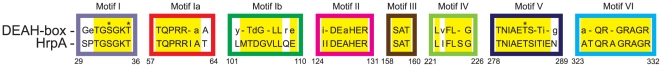
Schematic representation of conserved DEAH-box RNA helicase motifs in the *B. burgdorferi* HrpA protein. The sequence and location of conserved motifs of the DEAH-box family aligned with *B. burgdorferi* HrpA are shown. Amino acids in the DEAH-box consensus that are conserved at least 80% are shown in capital letters, and small letters represent the amino acids with 50–70% conservation. For further details regarding the DEAH-box motifs see [31], [32]. * denotes either T or S at three positions in the consensus sequence. The regions highlighted in yellow are perfect matches between the *B. burgdorferi* HrpA protein and the consensus sequence and the numbers below the boxes represents the position of the conserved motifs in HrpA.

As part of our ongoing work on antigenic variation in *B. burgdorferi*, we generated a disruption of the *hrpA (bb0827)* gene, which encodes a putative RNA helicase (**Fig. 1**), to see if loss of this function would have an effect upon antigenic switching at the *vlsE* locus. Surprisingly, the gene disruption resulted in a complete loss of infectivity and the modulation of the expression of about 180 *B. burgdorferi* proteins. Our findings suggest that HrpA is involved in a global regulatory pathway and may have relevance to regulation of virulence in other pathogens and to global regulatory mechanisms in bacteria in general.

## Results

### Construction of *hrpA* and *bb0826* gene disruptions in *B. burgdorferi*

**Figure 2:**
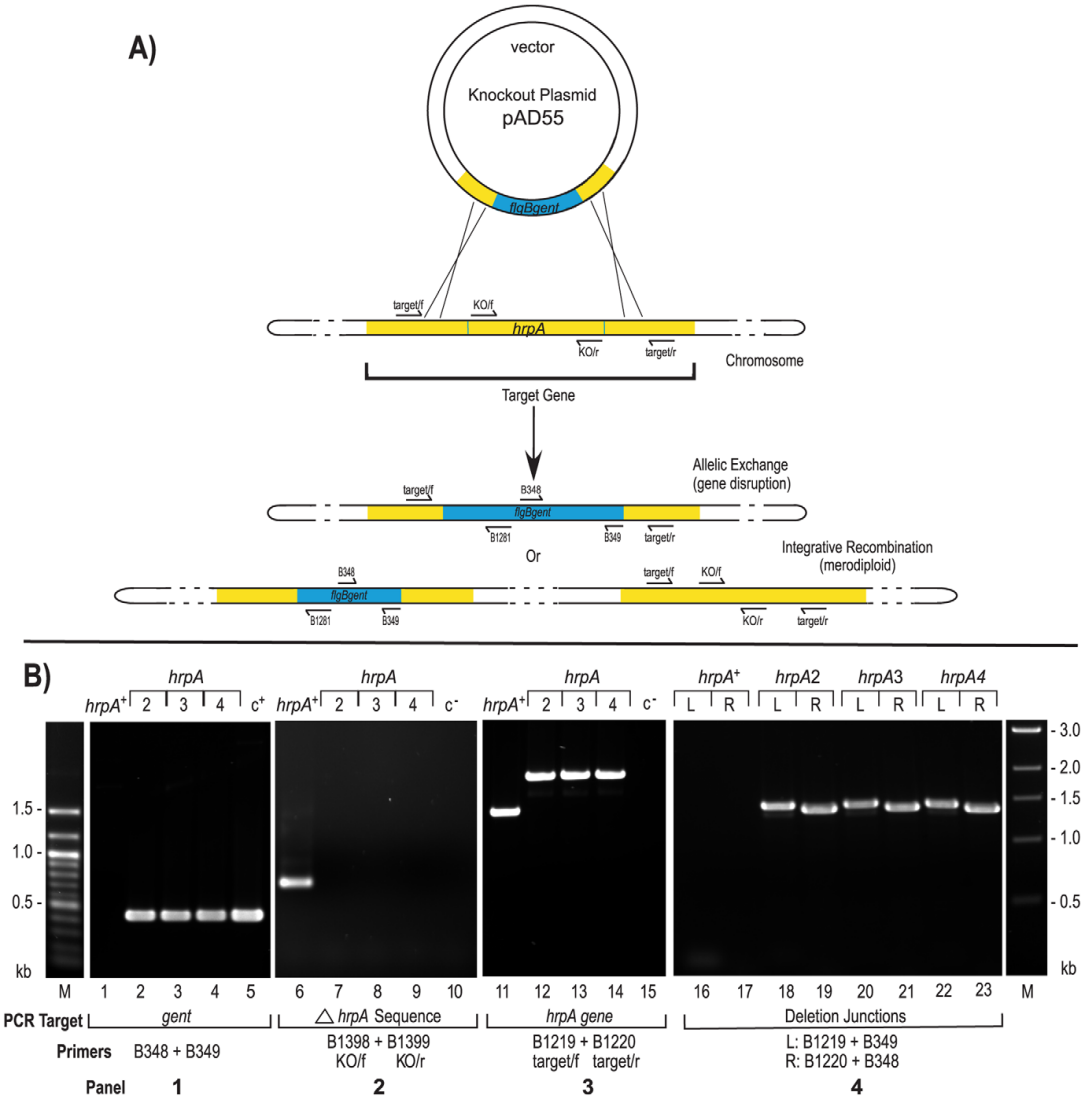
*hrpA* gene disruption and confirmation. **A**) Gene disruption strategy. The infectious *B. burgdorferi* strain B31, clone 5A4 (B31-5A4) was transformed with a knockout plasmid carrying a 1 kb gentamicin cassette (blue) that replaced the central 500 bp of the *hrpA* gene (yellow) as described in Materials and Methods. The two possible outcomes of recombination events with the target gene are shown: allelic exchange would result in gene disruption while integrative recombination of the knockout plasmid would result in merodiploid formation. The position of PCR primers used for construct verification are shown by arrows on the schematic. **B**) PCR verification of the *hrpA* disruption. Each gene disruption was subjected to four PCR analyses. **Panel 1**) The presence of the gentamicin resistance cassette was confirmed as shown. The shuttle vector pBSV2G [38] served as the positive control (c^+^) for amplification of the *gent* cassette (lane 5). **Panel 2**) The portion of *hrpA* expected to be deleted in a gene disruption was not detected in *hrpA2*, *3* or *4* (lanes 7, 8 and 9). Lane 10 was a negative control (c^−^) that lacked DNA template. **Panel 3**) The size of the *hrpA* gene was compared in the three mutant strains. The expected 2.1 kb gene disruption products were observed (lanes 12, 13 and 14) in comparison to the 1.5 kb product from the wild-type *hrpA* gene (lane 11). Lane 15 was a negative control (c^−^) that lacked DNA template. **Panel 4**) Confirmation of the correct insertion site was performed using combinations of the target gene primers and primers internal to the gentamicin cassette to amplify the *hrpA* boundaries. The left boundary in the *hrpA* knockout clones displayed the expected 1.4 kb product (lanes 18, 20 and 22) and the right boundary showed the expected product of approximately 1.3 kb (lanes 19, 21 and 23). A 100 bp ladder on the left side of Fig. 1B is relevant to the two left panels, and a 1 kb ladder on the right side applies to the two right panels (M). The schematic in part A of the figure is modified from [13].

Disruption of the *hrpA (bb0827)* gene in *B. burgdorferi* was accomplished by allelic exchange [36]. A knockout plasmid (**Fig. 2A**) was constructed in *E. coli*, in which the central 500 bp of the *hrpA* gene was deleted and replaced with a gentamycin resistance cassette (*aacC1*) under the control of the *B. burgdorferi flgB* promoter. The orientation of the resistance cassette relative to the target genes are shown in **Table 1**. A knockout plasmid was also constructed for *bb0826*, the gene downstream from *hrpA*, which contains an RNA binding motif and therefore, was considered as a protein that might function together with HrpA. The constructs were used to transform infectious *B. burgdorferi* B31 clone 5A4 [37]. The transformants recovered were screened by PCR as shown in **Fig. 2B** (results for *bb0826* not shown). First, the presence of the gentamycin resistance cassette was verified (Panel 1, lanes 2–4), followed by confirmation that the central 500 bp of the target gene was no longer present (panel 2, lanes7–9). To confirm that the recovered mutants carried only the disrupted *hrpA* gene and were not merodiploids, the presence of only the disrupted gene carrying the *gent* cassette (2.1 kb) was verified (Panel 3, lanes 12–14) along with the absence of the wild-type gene (1.5 kb, lane 11). Finally, the correct insertion site was confirmed using PCR primers to uniquely amplify left and right side deletion junctions (Panel 4, lanes 18–23). In addition, the structural integrity of the gene disruptions and the presence of only a single disrupted gene were independently demonstrated by Southern hybridization using probes specific for the gentamycin cassette and the deleted portion of the *hrpA* or *bb0826* gene (see **Fig. S1** for the Southern blot of the *hrpA* gene disruption).

**Table 1:** Plasmids and strains used in this study.

Gene target	Locus	Description	Plasmid	*E. coli* strains	*gent* polarity	*B. burgdorferi* mutant strains
*hrpA*	*bb0827*	RNA helicase	pAD55	GCE1567	forward	*hrpA2* (GCB1164), *hrpA3* (GCB1165), *hrpA4* GCB1166)
*bb0826*	*bb0826*	hypothetical prot	pPOH57-1	GCE2149	forward	*bb0826-11* (GCB544)
*bb0826*	*bb0826*	hypothetical prot	pPOH57-2	GCE2150	reverse	*bb0826-3* (GCB543)

All genetic constructs were analyzed for plasmid content, which can affect infectivity. No plasmid loss was observed for GCB1164 (*hrpA2*) and GCB1165 (*hrpA3*). GCB1166 (*hrpA4*) was lacking cp32-3 and cp32-6; cp32-3 is not required for infectivity [37] and the effect of loss of cp32-6 has not been previously reported. GCB543 (*bb0826-3*) and GCB544 (*bb0826-11*) both contained a full plasmid complement. Finally, analysis of transcription of the downstream genes *bb0825* and *bb0826* in the *hrpA* mutant strains was performed by RT-PCR to determine whether the *hrpA* gene disruptions had a polar effect and displayed reduced expression of the downstream genes; no decrease in downstream gene expression was observed in any of the three *hrpA* mutant clones (see **Fig. S2**). All three clones displayed wild-type morphology and normal growth in BSK-II media (data not shown).

### Effect of *hrpA* and *bb0826* gene disruptions on C3H/HeN mouse infections

The mutant strains were each used to infect C3H/HeN mice using an inoculum of 1×10^3^ spirochetes at two locations (see Materials and Methods). At seven days post-infection spirochetes were not recovered from the blood of any of the mice inoculated with the *hrpA* mutant clones, in contrast to the *bb0826* mutants and the control group where all the cultures were positive for spirochetes (**Table 2**). Similarly, ear cultures at day 21 were all negative for the mice inoculated with *hrpA* mutant spirochetes, but 100% positive for the *bb0826* mutants and the wild-type spirochetes. Finally, at day 35 when all cultures from heart, bladder, ear and joint were positive for wild-type and *bb0826* mutant *B. burgdorferi*, no positive cultures were recovered from the mice infected with the *hrpA* mutant clones. Mutation of the *hrpA* gene, therefore, appeared to obliterate spirochete infectivity. Attempts to complement the non-infectious phenotype by supplying the *hrpA* gene *in trans* on the shuttle vector pBSV2 [38] were unsuccessful (data not shown). Difficulty in complementing mutants in *B. burgdorferi* is not unusual and is elaborated upon in the Discussion.

**Table 2:** Effect of a mutation in the *B. burgdorferi hrpA (bb0827)* or *bb0826* gene on infection of C3H/HeN mice.

*B. burgdorferi* genotype	Strain	Total mice^a^	Day 7 Blood^b^	Day 7 Infection	Day 21 Ear	Day 21 Infection	Day 35^c^ Heart	Day 35^c^ Bladder	Day 35^c^ Joint	Day 35^c^ Ear	Total sites^d^	Day 35 Infection
B31 5A4 (wt)	GCB933	18	18/18	100.0%	18/18	100.0%	4/4	4/4	4/4	4/4	16/16	100.0%
*hrpA2*	GCB1164	3	0/3	0.0%	0/3	0.0%	0/3	0/3	0/3	0/3	0/12	0.0%
*hrpA3*	GCB1165	3	0/3	0.0%	0/3	0.0%	0/3	0/3	0/3	0/3	0/12	0.0%
*hrpA4*	GCB1166	3	0/3	0.0%	0/3	0.0%	0/3	0/3	0/3	0/3	0/12	0.0%
*bb0826-3*	GCB543	2	2/2	100.0%	2/2	100.0%	2/2	2/2	2/2	2/2	8/8	100.0%
*bb0826-11*	GCB544	2	2/2	100.0%	2/2	100.0%	2/2	2/2	2/2	2/2	8/8	100.0%

a There is a large number of mice in the control group because the *hrpA* mutants were initially assessed as part of a larger group of mutants.

b Values listed correspond to number of positive cultures/number of sites tested.

c Four mice infected with *B. burgdorferi* 5A4 were chosen as positive controls for organ harvests at day 35.

d Number of positive tissue sites/number of sites tested.

### 2D gel analysis of proteins from wild-type and *hrpA* mutant *B. burgdorferi*

**Figure 3:**
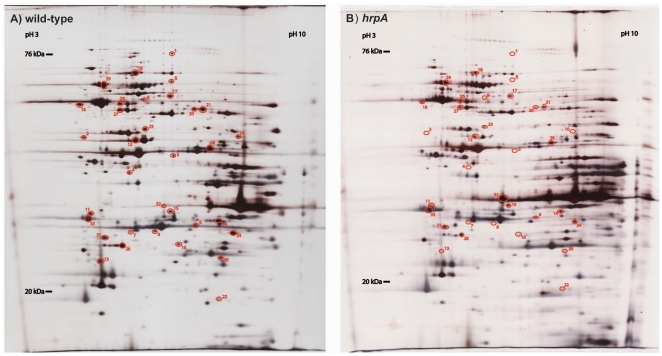
Silver stained 2D gel images. Whole cell protein extracts are shown for **A**) wild-type *B. burgdorferi* B31 clone 5A4 [37] and **B**) *hrpA* mutant strain GCB1165. Whole cell protein extracts were separated on non-linear pH 3–10 IPG strips in the first dimension and 12% SDS-PAGE gels in the second dimension. Differentially expressed protein spots are circled and numbered. Select proteins were identified by trypsin digestion followed by LC MS/MS analysis of spots from silver stained gels. **Table S3** shows relative fold differences in expression.

Because gene regulation by an RNA helicase is expected to occur primarily at the post-transcriptional level, we compared the protein content of wild-type *B. burgdorferi* B31 parent and their derivative *hrpA* mutant strains. Whole cell lysates were prepared from spirochetes grown to late log stage and were separated by two-dimensional gel electrophoresis. Silver staining methods were used to visualize proteins on the 2D gels. Approximately 600 protein spots were detected in the range of 10–80 kDa and the pH 3–10 area. The 2D gel profiles of the three *hrpA* mutant clones were indistinguishable from each other (data not shown). A comparison of the wild-type 2D gel profile compared to the *hrpA3* mutant (GCB1165) is shown in **Fig. 3**. In total, 33 protein spots were identified with changes in intensity between wild-type *B. burgdorferi* B31 and the *hrpA* mutant strains on silver stained analytical gels. Twenty of the proteins were identified by LC-MS/MS analysis of tryptic peptides coupled with a Mascot database search (**Table 3**). For 13 spots, the identification was not successful due to low MS signals or Mascot scores that were below the threshold. Both identified and unidentified protein spots are listed in decreasing order based upon their estimated fold decrease or increase relative to wild-type (**Table 3**). Out of the 20 identified spots, translation elongation factor Tu (EF-Tu, BB0476), and outer surface protein A (OspA, BBA15) were identified twice from different protein spots within one 2D gel, likely the result of protein modification. EF-Tu (spots 9 and 23) displayed the same migration in the isoelectric focusing dimension and ran with molecular masses of approximately 59 and 45 kDa, respectively. The 45 kDa spot corresponds to the full length protein and the higher mass band likely represents a modified form without any changes in pI. These two spots showed an estimated decrease of 8.27 and 2.62 fold in an *hrpA* background relative to wild-type. Interestingly, the OspA protein (spots 10 and 32) showed both a decrease of 6.81 (spot 10) and an increase of 8.01 fold (spot 32) in *hrpA* mutant strains. These two spots displayed vastly different migration in both dimensions, consistent with post-translational modification. Of the 18 proteins identified by 2D gels coupled with LC/MS-MS, 10 of these proteins were also identified by iTRAQ analysis (marked with asterisks in **Table 3**). The highest fold decrease (50.39) detected by 2D gels was from the large subunit of the putative phage terminase (BBM42). This protein is encoded by cp32-6, which was absent from one of the *hrpA* mutant clones used in this study, thereby contributing to an artificially high decrease in this protein relative to the wild-type strain. The average fold decrease from the two strains carrying cp32-6 was 34, still indicating a major change in expression.

**Table 3:** Changes in protein expression in *hrpA* mutant clones relative to wild-type *B. burgdorferi* as estimated by 2D gel electrophoresis.

Spot	ORF	Protein Name	Decrease
1	BB_M42	Phage terminase, large sub, pbsx fam	50.39
2	NA	NA	41.31
3	BB_N43	Phage terminase, large sub, pbsx fam	40.38
4	BB_0831	Xylose operon reg prot (XyIR-2)	32.84
5*	BB_0366	Vacuolar aminopeptidase I	32.62
6	NA	NA	31.64
7	NA	NA	22.64
8	NA	NA	11.58
9*	BB_0476	Translation elong factor Tu	8.27
10	BB_A15	Outer surface protein A (OspA)	6.81
11	BB_J02.1	Conserved hypothetical protein	6.21
12	NA	NA	6.02
13	NA	NA	4.94
14	BB_0658	Phosphoglycerate mutase fam prot	4.79
15*	BB_0215	ABC trans peripl PO_4_ bind prot (PstS)	4.24
16	BB_0239	Deoxyguan/deoxyadenosine kinase(Dck)	4.11
17	BB_0127	Ribosomal protein S1 (RpsA)	3.44
18	NA	NA	3.30
19*	BB_0540	Translation elong factor G	3.07
20	NA	NA	3.00
21*	BB_0020	PF6F1P β sub	2.84
22*	BB_0463	Nucleoside- diphosph kinase (NDK)	2.67
23*	BB_0476	Translation elong factor Tu	2.62
24*	BB_0375	Pfs protein (Pfs-1)	2.58
25*	BB_0513	Phe-tRNA ligase alpha chain (PheRS)	2.57
26	BB_0559	PTS system, gluce-spec IIA comp	2.48
27	NA	NA	1.87
28	NA	NA	1.71
29	NA	NA	1.58
30	NA	NA	1.53
31	NA	NA	1.52

Spot	ORF	Protein Name	Increase
32	BB_A15	Outer surface protein A (OspA)	8.01
33*	BB_J09	Outer surface protein D (OspD)	1.66

Changes in protein expression in *hrpA* mutants relative to wild-type *B. burgdorferi* as determined by 2D gel electrophoresis. Average fold increase or decrease was estimated as the mean value from two gels of the ratio between the spot volumes in wild-type *Borrelia burgdorferi* B31 and the *hrpA* mutant strains *hrpA2* (GCB1164), *hrpA3* (GCB1165) and *hrpA4* (GCB1166). Spot volumes were estimated using REDFIN 2D gel analysis software.

*indicates the proteins identified with both 2D gel and iTRAQ analysis.

NA indicates spots that were not identified. The 20 most abundant spots with a changed expression pattern in the mutants were identified by LC MS/MS analysis as described in materials and methods.

### iTRAQ proteome analysis for proteins differentially expressed in wild-type and *hrpA* mutant *B. burgdorferi*

Analysis by 2D gels is time consuming, only semi-quantitative, limited to analysis of single isoforms and requires gel spots that are resolvable as stained unique species. In contrast, iTRAQ analysis [39], [40] is expedient, allows simultaneous analysis of hundreds of proteins, is highly accurate, and captures data from all isoforms. To follow-up on the 2D gel results, quantitative iTRAQ analysis was performed on the *hrpA* mutant clones and the parental wild-type strain. For the iTRAQ proteome analysis, the goal was to create an experimental design that would allow control of experimental and technical variations as much as possible. Biological variation was controlled by pooling four independent cultures for each sample. In this way, protein from 32 cultures was analyzed in an 8-plex format. Protein extracts from the wild-type and each of the three mutant strains were analyzed in duplicate, where each of the eight samples was labeled with a different isobaric tag to allow simultaneous processing. Trypsinized proteins were analyzed and identified as described in Materials and Methods. Through the iTRAQ analysis, 370 proteins were identified (**Table S2**). Out of 370, 90 were significantly (P<0.05) downregulated in the *hrpA* mutant strains relative to the wild-type; 71 were encoded by the chromosome and 19 by plasmids (**Table S3**). An even greater number (97) of upregulated proteins were identified: 84 chromosomal and 13 plasmid-encoded (**Table S4**).

The fold decrease of the downregulated proteins was between 1.20 and 14.28 in an *hrpA* background. The downregulated proteins directly identified in this study were compared with genes previously reported to be regulated at the transcriptional level by microarray experiments [7], [12], [15], [21], [23], [24]. Out of 90 downregulated proteins identified in this study (**Table S3**), the RNA for four of them were reported as downregulated in an *rrp2* mutant while nine were upregulated in an *rrp2* background [21], [23]. In an *rpoN* mutant background four of the 90 were downregulated, 14 were upregulated and one was inconsistently reported as both down and up [7], [21]. In an *rpoS* mutant eight were downregulated and 10 were upregulated [7], [12], [21]. In an *rrp1* mutant 17 were downregulated and one was upregulated [24]. Finally, in a *bosR* mutant [15], the transcripts of seven genes were upregulated and none were downregulated. In summary, of the 90 proteins downregulated in *hrpA* mutant clones, the transcription for 42 of them showed no detectable regulation by any of the known *B. burgdorferi* regulators noted above.

The fold increase of the upregulated proteins was between 1.2 and 11.77 in an *hrpA* background. The upregulated proteins directly identified in this study were compared with genes previously reported to be regulated by microarray experiments [7], [12], [15], [21], [23], [24]. Out of 97 upregulated proteins identified in this study (**Table S4**), five of them have been reported as upregulated in an *rrp2* mutant while seven are downregulated in an *rrp2* background [21], [23]. In an *rpoN* mutant background nine of the 97 were upregulated and 11 were downregulated [7], [21]. In an *rpoS* mutant, nine were upregulated and 11 were downregulated [7], [12], [21], [23], [24]. In an *rrp1* mutant there were no upregulated proteins but eight were downregulated [24]. Finally, in a *bosR* mutant [15], the transcripts of three genes were upregulated and six were downregulated. In summary, of the 97 proteins upregulated in *hrpA* mutant clones, the transcription for 65 of them showed no detectable regulation by any of the known *B. burgdorferi* regulators noted above.

## Discussion

RNA regulation is a burgeoning field in bacteria in general [41], [42], [43], [44], [45], [46], [47] and in the control of virulence in bacterial pathogens [48], [49]. RNA helicases are ubiquitous proteins that play a role in a wide variety of RNA metabolic functions including transcription, ribosome biogenesis, RNA unwinding, RNA-protein complex disruption and reorganization, RNA processing and RNA decay [30], [31], [32], [33]. Based upon sequence analysis, *B. burgdorferi* appears to have a single RNA helicase, the DEAH-box protein HrpA (**Fig. 1**). In contrast, *E. coli* carries five DEAD-box helicases [50] as well as an HrpA orthologue [50]. HrpA from both *E. coli* [51] and *B. burgdorferi* (A. Salman-Dilgimen and G. Chaconas, unpublished) display ATPase activity *in vitro*. An *in vitro* helicase activity has not yet been reported for either protein, although the strong correspondence of the *B. burgdorferi* protein sequence to the eight motifs of ATP-dependent RNA helicases (**Fig. 1**) strongly supports assignment as an RNA helicase. Further biochemical characterization of HrpA will be required to conclusively establish an RNA helicase function.

During our studies on the proteins involved in antigenic switching at the *vlsE* locus of *B. burgdorferi* [13] we found that disruption of the *hrpA* gene resulted in complete loss of infectivity of C3H/HeN mice by needle inoculation (**Table 2**). This result was observed with three independent *hrpA* mutant clones, suggesting that the *hrpA* gene disruption was responsible for the phenotype, rather than some other defect introduced during genetic manipulation. To prove this point a wild-type *hrpA* gene with its native promoter (the *topA* promoter) was introduced into the mutant clones on an *E. coli – B. burgdorferi* shuttle vector, pBSV2 [38]. However, no restoration of infectivity was observed (data not shown). Genetic complementation in *B. burgdorferi* can sometimes be difficult to achieve. For example complementation of *ruvA* and *ruvB* using shuttle vectors could not be achieved in two independent laboratories [13], [52]. The reason for this remains unknown. We believe that the possibility of having a secondary mutation with an identical phenotype in infectivity and protein expression in all three of our clones is exceedingly low. We also showed that the *hrpA* gene disruption is not polar (**Fig. S2**) and that disruption of the downstream gene *bb0826* resulted in a completely infectious phenotype. From our combined data we conclude that the *hrpA* gene is required for infectivity, at least by needle inoculation and that HrpA is, therefore, a virulence determinant [53].

As a preliminary step in investigating the loss of infectivity we analyzed the protein content of our three *hrpA* mutant clones grown in culture, relative to the wild-type parent and found indistinguishable patterns in the three mutant strains with an obvious difference in protein expression relative to the wild-type parent strain (**Fig. 3** and **Table 3**). To more thoroughly and quantitatively investigate the differences in protein expression in the wild-type and mutant strains we performed a proteome analysis using iTRAQ methodology [39], [40]. We identified 370 *B. burgdorferi* proteins, of which 187 showed significant changes in expression compared to the wild-type parent: 90 were downregulated (**Table S3**) relative to wild-type and 97 were upregulated (**Table S4**). When compared to the changes observed with mutants in other regulatory systems, 42 of the 90 downregulated proteins (**Table S3**) and 65 of the 97 upregulated proteins (**Table S4**) are not regulated under any conditions by the previously reported *B. burgdorferi* regulators *bosR*, *rrp2*, *rpoN*, *rpoS* or *rrp1* [7], [12], [15], [21], [23], [24].

**Figure 4:**
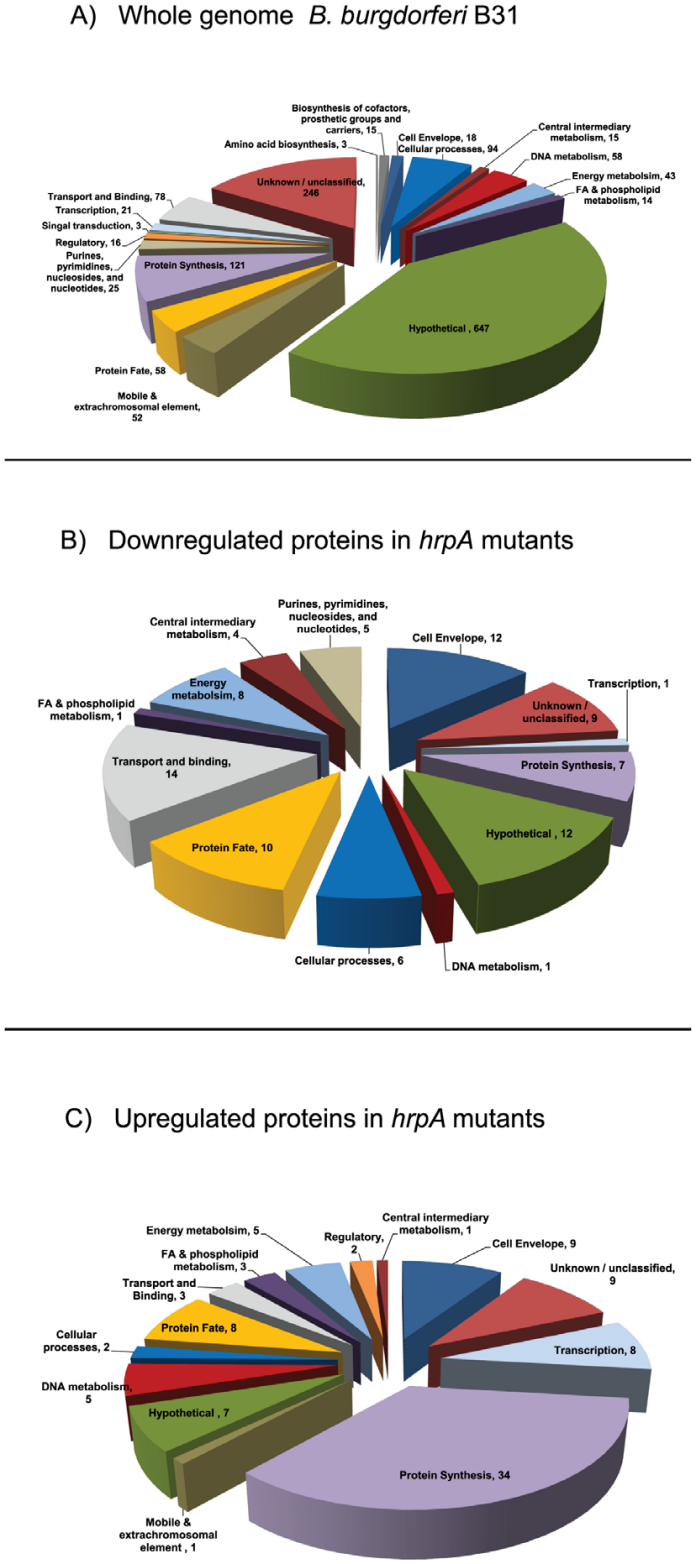
Pie chart representations for functional categorization of *B. burgdorferi* B31 proteins. **A**) The whole genome *B.burgdorferi* pie chart was created using data from The Comprehensive Microbial Resource database (http://cmr.jcvi.org/cgi-bin/CMR/shared/GetNumAndPercentGenesInARole.cgi). **B**) The pie chart showing proteins downregulated (total 90 proteins) in *hrpA* mutant clones was created using the data in **Table S3**. **C**) The pie chart showing proteins upregulated (total 97 proteins) in *hrpA* mutant clones was created using the data in **Table S4**.

Functional categorization of the regulated proteins relative to the *B. burgdorferi* proteome (**Fig. 4**) revealed that the most prominent downregulated categories were transport & binding, cell envelope, hypothetical proteins and protein fate. In contrast, the most prominent categories of upregulated proteins were protein synthesis, cell envelope, unclassified, transcription and protein fate, with protein synthesis representing 34% of the total. From the sum of the data discussed above we conclude that HrpA-mediated regulation is part of a new and distinct global regulatory pathway in *B. burgdorferi* gene expression. Since our iTRAQ analysis identified only a portion of the proteins in the total *B. burgdorferi* proteome, we have almost certainly identified only a subset of the HrpA-regulated *B. burgdorferi* proteins. Finally, it is noteworthy that HrpA itself has been reported to be slightly upregulated in an *rpoN* mutant [7].

Because an HrpA orthologue is present in many bacteria, its function in a global regulatory pathway in *B. burgdorferi* may have relevance in other bacterial species where its function remains obscure. The only known function of HrpA at this time is a single case where regulation of the expression of a fimbrial gene in *E. coli* has been reported [34]. Our work may, therefore, stimulate the investigation of possible global regulation of gene expression by HrpA in other bacteria. A role for an RNA helicase in global gene regulation in bacteria is both new and particularly timely with the recent growth of the field of RNA regulation of gene expression and the ability of RNA helicases to modulate RNA structure and function.

The reason why a complete loss of infectivity results from disruption of *hrpA* remains to be established. A notable point is that P66, which is required for infectivity, (Jenifer Coburn, personal communication) is downregulated five fold in the *hrpA* mutant and this may cause or contribute to the infectivity loss. In addition six of the 11 oligopeptide permeases (OppA-1, OppA-2, OppA-3, OppA-4, OppD and OppF) are all downregulated, which may compromise the ability of the spirochetes to survive in a mouse. Similarly, other transport systems were found to be downregulated in the *hrpA* strains (see **Table S3**) as were the protein chaperones GroEL and GroES. The inability to infect mice may result from a combined effect of the downregulation of these and other proteins. Alternatively, the upregulation of a variety proteins resulting from loss of *hrpA* may also have deleterious consequences for survival in the mouse. However, the experiments reported here have analyzed changes in protein expression during growth in culture. Therefore, we do not yet know the role of HrpA in the synthesis or stability of proteins in the tick vector or vertebrate host. Further studies will be required to assess the possibility of growing an *hrpA* mutant in ticks and in DMCs for such analyses. Growth of an *hrpA* mutant in ticks or DMCs would also aid further expression studies to be performed under different conditions to more thoroughly characterize the complete spectrum of HrpA regulated protein expression during the enzootic cycle.

In terms of the mechanisms by which HrpA might both upregulate and downregulate gene expression, it is too early to speculate. In the one case where HrpA has been shown to regulate fimbrial gene expression in *E. coli*, it is involved in the processing of a polycistronic mRNA [34]. HrpA may function in global regulation indirectly as a master regulator to turn on the expression of one or more activators or to turn off the expression of one or more repressors. We did not, however, uncover HrpA regulation of any of the known *B. burgdorferi* regulators such as BosR, Rrp2, RpoN, RpoS or Rrp1. Alternatively, HrpA might function directly in transcription or post-transcriptional events (see [30], [31], [32], [33]) for a large number of genes. The abundance of activities that can be modulated by RNA helicases, most of which are at the post-transcriptional level, leaves many possible mechanistic alternatives. Further studies on HrpA regulation *in vivo* as well as protein function *in vitro* will help to elucidate the mechanism of this fascinating protein, which may possibly hold a general role in global regulation of gene expression in bacteria.

## Materials and Methods

### Ethics statement

This study was carried out in accordance with the principles outlined in the most recent policies and *Guide to the Care and Use of Experimental Animals* by The Canadian Council on Animal Care. Our animal protocol (M08042) was approved by The Animal Care Committee of the University of Calgary.

### Bacterial strains and culture conditions

*E. coli* DH5α was used for the construction and maintenance of the knockout plasmids. Infectious *Borrelia burgdorferi* B31 clone 5A4 [37] was used as the parental strain to generate the *hrpA* and *bb0826* mutants. All *B. burgdorferi* clones were cultivated at 35°C (with a 1.5% CO_2_ environment) in BSK-II medium prepared in-house [54] and supplemented with 6% rabbit serum (Cedarlane Laboratories, Burlington, ON, Canada). To cultivate *B. burgdorferi* from mouse tissues, 1× *Borrelia* antibiotic cocktail (20 µg/ml phosphomycin, 50 µg/ml rifampicin and 2.5 µg/ml amphotericin B) was added to the culture media. Bacterial density was determined by dark-field microscopy using a Petroff-Hausser chamber.

### Gene disruption in *B. burgdorferi*

Disruption of *hrpA* and *bb0826* was performed as previously described [13]. Briefly, a 1.5 kb region containing the targeted gene was amplified from *B. burgdorferi* B31 clone 5A4 DNA [37] by PCR using target/f and target/r primers (see **Table S1**) and cloned into either pJET1.2/Blunt (Fermentas) for *hrpA* or pCR-BluntII-TOPO (Invitrogen) for *bb0826*. The central part of the target gene was removed by inverse PCR using outward-oriented primers containing a 5′ NheI restriction site and replaced by a gentamicin resistance cassette (*aacC1*) under the control of the *flgB* promoter. For the *bb0826* knockout plasmid, the gentamicin resistance cassette was amplified from pBSV2g [55] using B415 and B416 primers containing 5′ NheI restriction site. For the disruption of the *hrpA* gene, the *flgB* promoter-driven gentamicin resistance cassette was fused to a T7 transcriptional terminator ( *5′ CTG CTA ACA AAG CCC GAA AGG AAG CTG AGT TGG CTG CTG CCA CCG CTG AGC AAT AAC TAG CA TAA CCC CTT GGG GCC TCT AAA CGG GTC TTG AGG GGT TTT TTG 3′* ) by overlap extension PCR. Resistance to gentamicin was used as the selectable marker. The orientation of the gentamicin resistance cassette relatively to the target gene was determined by PCR using either target/f or target/r primer, specific to the gene target, in combination with either B348 or B349 for the *hrpA* disruption or in combination with B348 or B1281 for the *bb0826* knockout (See **Figure 2** and **Table 1**). To generate *hrpA* and *bb0826* gene disruptions, each knockout plasmid was used to transform infectious *B. burgdorferi* B31 clone 5A4 strain by electroporation. Gene disruption was confirmed by PCR and Southern blot hybridization as previously described [13], [56], [57] and as described in the Results section. Plasmid content was analyzed as previously described [13] and three isolates were chosen for further analysis. The three isolates were classified as independent clones based upon subsequent transformability and plasmid content.

### RT-PCR analysis for the downstream genes of *hrpA*; *bb0825* and *bb0826*

*B. burgdorferi* cultures were harvested by centrifugation when they reached the concentration of ∼1×10^8^ cells/ml in 10 ml BSK-II medium prepared in-house. RNA was extracted using an Aurum Total RNA Mini Kit as per manufacturer's instructions. RNA concentrations were determined using a NanoDrop spectrophotometer and the integrity of the RNA was assessed by agarose gel electrophoresis. cDNA was generated for *bb0825* and *bb0826* by The RevertAid H Minus First Strand cDNA Synthesis Kit, Fermentas using gene specific primers (**Table S1**) according to manufacturer's instructions. For subsequent PCR reactions, 1 µl of cDNA was used as a template in 50 µl reactions with 10 pmoles of each primer. PCR reactions were run for 25, 30 and 35 cycles to ensure that saturation had not been reached.

### Mouse infections

Three to four week old male C3H/HeN wild-type mice were obtained from Harlan Laboratories or Charles River Laboratories (St-Constant, QC). Mice were infected by intraperitoneal and dorsal subcutaneous injection of 100 µl containing 1×10^4^ spirochetes/ml at each site. Infectivity and persistence of *B. burgdorferi* in mice was determined as previously described [13]. A 50 µl blood sample was collected from the saphenous vein seven days post-infection and cultivated as described above. At day 14 and 21, two ear punch biopsies were taken and cultivated for the presence of spirochetes. The heart, ear, bladder and joint were collected 35 days post-infection and cultivated. The presence of *B. burgdorferi* in culture was determined by dark-field microscopy.

### Protein sample preparation

For 2D gel electrophoresis wild-type *B. burgdorferi* B31 clone 5A4 [37] and the *hrpA* mutant strains GCB1164 (*hrpA2*), GCB1165 (*hrpA3*) and GCB1166 (*hrpA4*) were grown to late log phase (∼1×10^8^ cells/ml) in 100 ml BSK-II medium prepared in-house and harvested by centrifugation (8000× g, 15 min, 4°C). Cell pellets were washed twice with 50 ml of 50 mM Tris-HCl, pH 7.5 and centrifuged as above. The pellets were then suspended in lysis buffer containing 25 mM Tris-Base, 50 mM KCl, 3 mM EDTA, 3 mM, benzamidine, 2.1 µM leupeptine, 9 M Urea, 2% ampholyte 3–10, 1% TritonX-100, 70 mM DTT and protease inhibitor cocktail (according to the manufacturer's instructions, Sigma-Aldrich, Cat No. P8465). The cells were lysed by 5 cycles of freezing in liquid nitrogen and thawing at room temperature. The soluble whole cell protein extracts were collected by centrifugation at 50,000× g at room temperature for 45 minutes. The samples were then reduced by addition of tributylphosphine to 5 mM at room temperature with an incubation of one hour with occasional shaking. The samples were subsequently alkylated by addition of iodoacetamide to 15 mM with an incubation of 1.5 hours at room temperature in the dark.

For iTRAQ, samples were harvested and prepared as noted above, with some modifications. For iTRAQ, DTT was not included in the lysis buffer and the reduction and alkylation steps were excluded from the sample preparation method. Samples were then diluted with HPLC-grade H_2_O to a final concentration of 4 M urea and then precipitated overnight with ice-cold acetone at −20°C. Precipitated samples were collected by centrifugation at 8,000× g at 4°C for 20 minutes and the pellets were dried at room temparature for 30 minutes. Dried samples were submitted for analysis at the University of Victoria Genome BC Proteomics Center.

### 2D gel electrophoresis experiments and protein identification

Protein concentrations were determined using the Bradford protein assay [58] with BSA as a standard. For separation in the first dimension by isoelectric focusing (IPG gels, GE Healthcare Immobiline™ DryStrip, pH 3–10 NL, 18 cm) samples (150 µg for analytical and 250 µg for preparative gels) were applied on strips allowing an overnight rehydration in buffer containing 8 M urea, 2% (w/v) Triton X-100, 0.5% (v/v) ampholyte 3–10, 0.002% Bromophenol blue and 18 mM DTT. The strips were focused using the IPGphor system (Amersham) with a gradient voltage increase of 500 V for 1 hour, 1000 V for 8 hours, 8000 V for 3 hours and a constant 8000 V for 2.5 hours. The focused strips were stored at −80°C until the second dimensional run.

Prior to the second dimensional separation by 12% SDS PAGE, the strips were equilibrated sequentially in two different buffers. The strips were first incubated in equilibration buffer I containing 6 M urea, 75 mM Tris-HCl pH 8.8, 29% (v/v) glycerol, 2% SDS (w/v), 0.002% bromophenol blue and 64 mM DTT with a gentle shaking, for 15 minutes at room temperature. Following this, the strips were incubated in equilibration buffer II containing 6 M urea, 75 mM Tris-HCl pH 8.8, 29% (v/v) glycerol, 2% SDS (w/v), 0.002% bromophenol blue and 135 mM iodoacetamide for a further 15 minutes at room temperature with gentle shaking. Second dimensional runs were standard 12% SDS-PAGE gels (18 cm×20 cm). The runs were started at 90 V for one hour, followed by 160 V for one hour and 200 V for five hours.

Analytical 2D gels were silver stained [59], [60] (Heukeshoven method for analytical and Shevchenko method for preparative gels) and analytical gels were dried under vacuum in a gel dryer. Protein expression differences between wild-type *B. burgdorferi* B31 clone 5A4 and the *hrpA* mutant strains GCB1164, GCB1165 and GCB1166 were compared to determine differential expression using REDFIN 2D gel analysis software (Ludesi, Sweden). Protein abundance changes of 1.5 or greater for the average of the three mutant clones versus the wild-type were candidates for differentially expressed proteins and were circled and numbered in Fig. 3. Some of these spots were cut out from silver stained preparative gels for in-gel tryptic digestion and subsequent identification by LC- MS/MS at Southern Alberta Mass Spectrometry (SAMS) Centre for Proteomics, a Core Facility of the University of Calgary, Faculty of Medicine using a MASCOT (Matrix science Ltd., London United Kingdom, www.matrixscience.com) database search. For in-gel tryptic digestion, briefly, protein spots were cut out from preparative silver stained gels with a scalpel, minced into one mm^3^ pieces and transferred to pre-washed (60% Acetonitrile/0.1% TFA) microcentrifuge tubes. Spots were washed two times with HPLC-grade H_2_O for 10 minutes and then with freshly made destaining solution containing 30 mM K_3_Fe(CN)_6_ and 100 mM Na_2_S_2_O_3_, 2 times for eight minutes at room temperature. After destaining a second H_2_O wash was performed. Excessive washing and short periodic low speed vortexing were done when necessary till all the stain was removed. Following this, dehydration of the gel pieces was performed with an incubation in 100% acetonitrile for 10 minutes at room temperature. All the acetonitrile was then removed and opaque gel pieces were air-dried at room temperature and then rehydrated for 60 minutes at 4°C in 20–50 µl of trypsin working solution (50 mM ammonium bicarbonate, 10 mM CaCl_2_, 1% acetonitrile and 20 ng/µl trypsin, Princeton Separations, porcine, sequencing grade, modified). Samples were then incubated at 37°C overnight. The next day 1/5 volume 5% TFA was added to the samples followed by incubation at 60°C for 1 hour. Digested samples were then cleaned up using Zip Tips C18 (Millipore) and eluted with 10 µl elution solution (85% acetonitrile, 0.1% TFA). Eluted samples were stored at −20°C for further LC MS/MS analysis.

### iTRAQ experimental design and analysis

The iTRAQ analysis was designed as an 8-plex experiment enabling two comparisons of wild-type *B. burgdorferi* to three separate *hrpA* mutant clones. To control for biological variation, 8 individual samples (B31-1, B31-2, 1164-1, 1164-2, 1165-1, 1165-2, 1166-1, and 1166-2) were prepared by pooling four independent cultures for each. Technical and experimental variations were controlled by having replicate samples (e.g. B31-1 and B31-2) digested separately and tagged with different isobaric tags in the 8-plex iTRAQ experiment.

Total protein extracts were submitted to the University of Victoria-Genome BC Proteomics Centre for iTRAQ analysis. Briefly, samples were reduced with TCEP (Tris[2-carboxyethyl] phosphine) and alkylated with MMTS (s-methyl thiomethanesulfonate). Proteins were then digested in solution with trypsin (Promega) and labeled with appropriate iTRAQ labels. Isobaric labels assigned to individual samples were as follows; B31-1/113, 1164-1/114, 1165-1/115, 1166-1/116, B31-2/117, 1164-2/118, 1165-2/119, 1166-2/121. The labeled peptides were combined and separated by strong cation exchange HPLC. Fractions were then analyzed by LC-MS/MS.

### LC-MS/MS

LC-MS/MS analysis was performed using an integrated Famos autosampler, Switchos II switching pump, and UltiMate micro pump (LC Packings, Amsterdam) system with an Hybrid Quadrupole-TOF LC/MS/MS Mass Spectrometer (QStar Pulsar i) equipped with a nano-electrospray ionization source (Proxeon, Odense, Denmark) and fitted with a 10 µm fused-silica emitter tip (New Objective, Woburn, MA). Chromatographic separation was achieved on a 75 µm×15 cm C18 PepMap Nano LC column and a 300 µm×5 mm C18 PepMap guard column was in place before switching inline with the analytical column and the MS. The mobile phase (solvent A) consisted of water/acetonitrile (98∶2 (v/v)) with 0.05% formic acid for sample injection and equilibration on the guard column at a flow rate of 100 µl/min. A linear gradient was created by mixing solvent A with solvent B that consisted of acetonitrile/water (98∶2 (v/v)) with 0.05% formic acid at a reduced flow rate of 200 nl/min for high resolution chromatography and introduction to mass spectrometer.

Samples were brought up to 100 µl with 5% acetonitrile and 3% formic acid and transferred to autosampler vials (LC Packings, Amsterdam). 20 µl of sample were injected in 95% solvent A and allowed to equilibrate on the trapping column for 10 mins. Upon switching inline with the MS, a linear gradient from 95% to 40% solvent A was developed for 100 minutes and in the following 5 minutes the composition of the mobile phase was decreased to 20% solvent A before increasing to 95% solvent A for a 15 minute column re-equilibration prior to the next sample injection.

MS data was acquired automatically using Analyst QS 1.0 software Service Pack 8 (ABI MSD SCIEX, Concord, Canada). An information dependent acquisition method consisting of a 1 second TOFMS survey scan of mass range 400–1200 amu and two 2.5 second product ion scans of mass range 100–1500 amu. The two most intense peaks over 20 counts, with charge state 2–5 were selected for fragmentation and a 6 amu window was used to prevent the peaks from the same isotopic cluster from being fragmented again. Once an ion was selected for MS/MS fragmentation it was put on an exclude list for 180 seconds. Curtain gas was set at 23, nitrogen was used as the collision gas and the ionization tip voltage used was 2700 V.

All data files were searched for protein identification and relative abundance using Protein Pilot (V.3.0) and were searched against a custom *B. burgdorferi* B31 database. A positive protein identification required at least two matching peptide sequences with a minimum of a 95% confidence limit in ProtScore.

iTRAQ ratios were expressed as mutant/wild-type, using wild-type B31-1 and B31-2 samples (isobaric labels 113 and 117, respectively) as denominators in Protein Pilot software. Finally, the 12 iTRAQ ratios obtained (see **Table S2**) were used for subsequent data analysis. After transferring all data sets from Protein Pilot to Microsoft Excel, iTRAQ values were ranked according to the mean values calculated from 12 individual ratios. P values were calculated based upon the means of the 12 input ratios using a two-sided, one sample *t*-test with comparison against a theoretical value of 1.0 using GraphPad Instat (V.3.10). iTRAQ ratios ≥1.2 or ≤0.8 were considered as being differentially expressed if the P values were ≤0.05. Downregulated and upregulated proteins were grouped separately in **Table S3** and **Table S4**, respectively. The total list of iTRAQ-identified proteins is shown in **Table S2**.

## Supporting Information

Figure S1 **Gene disruption and the absence of additional copies of the** ***hrpA*** **gene was confirmed by Southern hybridization.** Genomic DNA was digested with HindIII and run on a 1.0% agarose gel with a 1 kb molecular weight ladder (M). Probes complementary to the gentamicin (*gent*) resistance cassette (left panel) and the portion of the *hrpA* gene deleted during gene disruption (right panel) were used for hybridization to duplicate blots. As expected, hybridization to the *gent* probe was not observed in the wild-type strain but was observed at the expected size (7.1 kb) for the three *hrpA* mutant strains. Conversely, hybridization to the deleted portion of *hrpA* was observed in the expected 6.5 kb fragment in the wild-type strain but not in the three *hrpA* mutant clones.
(EPS)

Figure S2 **Region view and transcription patterns of** ***hrpA*** **,** ***bb0825*** **and** ***bb0826*** **.** **A**) Schematic representation of *hrpA* and the two downstream genes on the *B. burgdorferi* chromosome. Arrows represent the direction of transcription and the numbers denote the coordinates on the chromosome. **B**) Ethidium bromide stained 1.4% agarose gel showing the products of RT-PCR reactions to assess the transcription patterns of genes *bb0825* and *bb0826* in the three *hrpA* mutant clones, along with a 100 bp molecular weight ladder (M). **Panel 1**) RT-PCR reactions for *bb0825* in the wild-type parent strain (*B. burgdorferi* B31, clone 5A4) and in the mutants *hrpA2*, *3* and *4*. The expected product size was 210 bp. **Panel 2**) RT-PCR reactions for *bb0826* in the strains described in Panel 1. The expected product was 310 bp.
(EPS)

Table S1 Primers used in this study.
(PDF)

Table S2 Complete listing of iTRAQ results.
(PDF)

Table S3 Downregulated *B. burgdorferi* proteins in *hrpA* mutant clones compared to wild-type based upon iTRAQ analysis.
(PDF)

Table S4 Upregulated *B. burgdorferi* proteins in *hrpA* mutant clones compared to wild-type, based upon iTRAQ analysis.
(PDF)
