# Supplementary material for: HrpA, a DEAH-Box RNA Helicase, Is Involved in Global Gene Regulation in the Lyme Disease Spirochete
Source: PLoS One. 2011 Jul 26;6(7):e22168. doi: 10.1371/journal.pone.0022168 (PMC3144200; doi:10.1371/journal.pone.0022168)
Supplement: Table S1 — Primers used in this study. (PDF) [file pone.0022168.s003.pdf]

Table S1. Primers used in this study

**Primers used for knockout plasmid construction and screening**			
**Genotype**	**Target (f/r)**	**Inverse**	**Knockout (KO f/r)**
hrpA	B1219f-GTTATTTTTGTATTCCGCTTT	B1229-ATGCAATCCGCTAGCAAAAGCTCTAAATAAAGAAGCTT	B1398-TTAAAATCTTCAAAGATATTAACAA
	B1220r-TTCGTCTGCTACAATAAACAC	B1230-ATGCAATCCGCTAGCTTGAGTTGCTGATGATTTT	B1399-GCAGGAAGACTTTCAAAA
bb0826	B1845f-GCATAAAAATATAACGGTTTTTAACA	B1848-GCGCTAGCTAGCTAGGTTCATAATTTATTATAAACTTCATTGCTG	B1876-GGCTATCCTTTTTTTATGTTTTATT
	B1846r-CCTATTATTGTTCTTGGGTGC	B1849-GCGCTAGCTAGCTAGGATGTCAAAATAATTTATCAAATATTCAG	B1877-CTTCATTTATTCTTAATTTTAGCG
**Primers used for *gent* cloning and transformant screens**			
**Purpose**	**Primer**		
Screening	B348-CGCAGCAGCAACGATGTTAC		
Screening	B349-CTTGCACGTAGATCACATAAGC		
Screening	B1281-ATCGTCTATGCTTAAGCTCTT		
Cloning	B415-CATTTCCTAGCTAGCGGCGAATGGCGCGGCCGCCCTAGG		
Cloning	B416-CATTTCCTAGCTAGCACGCGTAAGCCGATCTCGGCTTG		
Cloning	B820-CATTTCCTAGCTAGCCCTAGGTAATACCCGAGCTTCAA		
Cloning	B1345-CATTTCGCTAGCCAGATCCGGATATAGTTCCTCCTTTC		
Cloning	B1349-CCCAAGTACCGCCACCTAATCAGAATTGGTTAATTGGTTGTAACACT		
Cloning	B1350-AGTGTTACAACCAATTAACCAATTCTGATTAGGTGGCGGTACTTGGG		
**Primers used for RT-PCR of *bb0825* and *bb0826***			
**Purpose**	**Primer**		
bb0825 RT	B1762-CACTGCTAGTATTGATTTTAAG		
bb0825 For	B1755-CTACGGAGAGATTGTAATAAAGC		
bb0825 Rev	B1756-GACACCCCTTCCTTCTATTG		
bb0826 RT	B1763-GCTACATATGCTTTATGATTTAACC		
bb0826 For	B1757-GACTTAGAACAACATTATCTGCTAATTAC		
bb0826 Rev	B1758-GAATAAATGCTTTTGGGATAGTTTG		
