# Supplementary material for: HrpA, a DEAH-Box RNA Helicase, Is Involved in Global Gene Regulation in the Lyme Disease Spirochete
Source: PLoS One. 2011 Jul 26;6(7):e22168. doi: 10.1371/journal.pone.0022168 (PMC3144200; doi:10.1371/journal.pone.0022168)
Supplement: Table S2 — Complete listing of iTRAQ results. (PDF) [file pone.0022168.s004.pdf]

Table S2. Complete listing of iTRAQ results

N	ORF	Protein Name	114:113	118:113	114:117	118:117	115:113	119:113	115:117	119:117	116:113	121:113	116:117	121:117	Mean ± SD	P value
79	BB_0241	glycerol kinase (glpK)	0.0895	0.053	0.0946	0.056	0.1191	0.0839	0.1259	0.0879	0.0409	0.0299	0.0417	0.031	0.07±0.03	0.0001
148	BB_0243	glycerol-3-phosphate dehydrogenase anaerobic (glpA)	0.1067	0.0817	0.1117	0.0855	0.1472	0.118	0.1542	0.1247	0.1009	0.1107	0.1057	0.1148	0.11±0.02	0.0001
11	BB_0603	membrane-associated protein p66	0.2188	0.2377	0.2014	0.2168	0.2128	0.2858	0.1923	0.2582	0.1905	0.0631	0.1738	0.0592	0.19±0.07	0.0001
82	BB_A74	outer membrane porin (oms28)	0.3837	0.4365	0.4875	0.5495	0.035	0.0506	0.0394	0.0625	0.2754	0.1472	0.3467	0.182	0.25±0.18	0.0001
10	BB_0330	oligopeptide ABC (oppA-3)	0.2992	0.4018	0.2965	0.3945	0.2377	0.2831	0.2312	0.2754	0.4246	0.1282	0.4169	0.1247	0.29±0.1	0.0001
46	BB_B29	PTS system maltose and glucose-specific IIABC component (malX)	0.2729	0.278	0.3076	0.3251	0.3597	0.3565	0.4018	0.3981	0.2512	0.0929	0.2858	0.1009	0.29±0.1	0.0001
44	BB_0365	lipoprotein LA7	0.4831	0.3767	0.5445	0.4285	0.278	0.2148	0.3162	0.2399	0.2831	0.0619	0.3281	0.0679	0.3±0.14	0.0001
255	BB_B07	outer surface protein putative	0.1871	0.1542	0.2014	0.2208	0.3873	0.3499	0.5152	0.4613	0.2399	0.3373	0.3162	0.4446	0.32±0.11	0.0001
16	BB_0328	oligopeptide ABC transporte (oppA-1)	0.2512	0.2559	0.2228	0.2208	0.5916	0.4055	0.5152	0.3532	0.5649	0.1225	0.492	0.1076	0.34±0.16	0.0001
17	BB_0337	enolase (eno)	0.3981	0.3698	0.3802	0.3467	0.3404	0.4571	0.3162	0.4325	0.3373	0.2679	0.3192	0.2559	0.35±0.06	0.0001
75	BB_0238	predicted coding region BB0238	0.4246	0.4613	0.4055	0.4487	0.3565	0.4699	0.3436	0.4487	0.4207	0.1514	0.4055	0.1459	0.37±0.11	0.0001
25	BB_J09	outer surface protein D (ospD)	0.3467	0.2858	0.3105	0.2535	0.3565	0.4446	0.3162	0.3981	0.8017	0.1923	0.7112	0.1722	0.38±0.18	0.0001
22	BB_0476	translation elongation factor TU (tuf)	0.3664	0.5861	0.3837	0.5754	0.4406	0.4656	0.4365	0.4699	0.1738	0.3342	0.1738	0.3467	0.4±0.13	0.0001
9	BB_0744	antigen p83/100	0.227	0.3565	0.2377	0.3499	0.6368	0.631	0.6252	0.6194	0.4093	0.2128	0.4018	0.207	0.41±0.17	0.0001
470	BB_B06	PTS system cellobiose-specific IIB component (celA)	0.4613	0.4055	0.4656	0.4055	0.3664	0.5012	0.3698	0.5012	0.4018	0.4571	0.4055	0.4613	0.43±0.04	0.0001
263	BB_0622	acetate kinase (ackA)	0.5395	0.5754	0.6918	0.7379	0.2489	0.3733	0.3076	0.4742	0.3767	0.2188	0.4656	0.2606	0.44±0.16	0.0001
35	BB_0210	surface-located membrane protein 1 (lmp1)	0.2965	0.6081	0.2704	0.4966	0.5105	0.6194	0.4055	0.4966	0.7311	0.2228	0.597	0.182	0.45±0.17	0.0001
87	BB_I29	predicted coding region BBI29	0.4529	0.3981	0.4365	0.3837	0.6252	0.597	0.6026	0.5754	0.6194	0.1057	0.597	0.1009	0.46±0.18	0.0001
365	BB_B05	PTS system cellobiose-specific IIA component (celC)	0.863	0.7379	0.2333	0.1977	0.3251	1.0375	0.0871	0.278	0.7178	0.7727	0.1923	0.207	0.47±0.31	0.0002
174	BB_0408	PTS system fructose-specific IIABC component (fruA-1)	0.492	0.4446	0.4966	0.4487	0.3076	0.7656	0.3076	0.7656	0.5445	0.302	0.5546	0.2858	0.48±0.16	0.0001
23	BB_0329	oligopeptide ABC transporter (oppA-2)	0.3767	0.6427	0.3048	0.52	0.5808	0.6026	0.4656	0.4831	0.7943	0.2679	0.6368	0.2188	0.49±0.17	0.0001
31	BB_0020	pyrophosphate-fruc. 6-P -1-phosphotransferase beta sub (pfpB)	0.3733	0.7112	0.3373	0.631	0.7178	0.6194	0.6368	0.5495	0.2466	0.4446	0.2168	0.4018	0.49±0.17	0.0001
113	BB_0204	Lambda CII stability-governing protein (hflC)	0.7178	0.5346	0.5808	0.4406	0.7047	0.5861	0.5702	0.4786	0.4966	0.1905	0.4093	0.1644	0.49±0.17	0.0001
37	BB_0385	basic membrane protein D (bmpD)	0.3565	0.5702	0.4529	0.7047	0.5916	0.5916	0.7311	0.7311	0.2655	0.2729	0.3404	0.3499	0.5±0.17	0.0001
244	BB_0517	heat shock protein (dnaJ-1)	0.3192	0.4613	0.3342	0.4786	0.4529	0.6546	0.4786	0.6918	0.6138	0.4742	0.6607	0.5012	0.51±0.12	0.0001
29	BB_0383	basic membrane protein A (bmpA)	0.5861	0.7311	0.4285	0.52	0.6855	0.7112	0.492	0.52	0.6918	0.1722	0.5105	0.1247	0.51±0.19	0.0001
124	BB_0426	predicted coding region BB0426	0.2754	0.7656	0.2704	0.7516	0.6026	0.7178	0.5916	0.7047	0.6486	0.0938	0.6368	0.0938	0.51±0.24	0.0001
153	BB_K47	predicted coding region BBK47	0.8166	0.6668	0.7943	0.6546	0.5649	0.3664	0.5546	0.3597	0.6081	0.1169	0.597	0.1138	0.52±0.22	0.0001
2	BB_0649	heat shock protein (groEL)	0.2655	0.4246	0.2466	0.3597	0.9638	1	0.7943	0.8318	0.5808	0.1675	0.4831	0.1472	0.52±0.29	0.0002
48	BB_0323	predicted coding region BB0323	0.5808	0.5754	0.5346	0.5297	0.5702	0.5754	0.5297	0.5346	0.6026	0.4656	0.5546	0.4325	0.54±0.05	0.0001
64	BB_0608	aminoacyl-histidine dipeptidase (pepD)	0.5861	0.5445	0.6855	0.6427	0.6427	0.5105	0.7656	0.6026	0.4246	0.2831	0.4966	0.3342	0.54±0.14	0.0001
102	BB_0659	lysyl-tRNA synthetase	0.4246	0.5808	0.5445	0.7244	0.6427	0.597	0.7943	0.7379	0.5495	0.2249	0.673	0.2655	0.56±0.17	0.0001
328	BB_0462	conserved hypothetical protein	0.5861	0.7379	0.4613	0.5808	0.5598	0.5916	0.4446	0.4656	0.8472	0.5248	0.6668	0.4169	0.57±0.12	0.0001
68	BB_0375	pfs protein (pfs-1)	0.6982	0.879	0.6081	0.7656	0.4365	0.492	0.3908	0.4446	0.3981	0.7379	0.3565	0.6427	0.57±0.17	0.0001
325	BB_0024	predicted coding region BB0024	0.5808	0.6138	0.4406	0.4699	1.0471	0.5598	0.8017	0.4285	0.7047	0.4571	0.5395	0.3467	0.58±0.18	0.0001
43	BB_0668	flagellar filament outer layer protein (flaA)	0.8872	0.8472	0.6194	0.597	0.8395	0.7798	0.5754	0.5445	0.6252	0.1225	0.4365	0.0817	0.58±0.25	0.0002
74	BB_0536	zinc protease putative	0.4529	0.5754	0.5105	0.6546	0.5297	0.631	0.597	0.7178	0.4699	0.7379	0.5395	0.8395	0.6±0.11	0.0001
80	BB_0741	chaperonin (groES)	0.7311	0.7447	0.597	0.5861	0.5546	0.7727	0.4529	0.6081	0.9376	0.278	0.7379	0.2168	0.6±0.2	0.0001
100	BB_0334	oligopeptide ABC transporter ATP-binding protein (oppD)	0.7112	0.5248	0.7516	0.5754	0.5248	0.492	0.5702	0.5395	0.8091	0.4613	0.879	0.4966	0.61±0.13	0.0001
18	BB_B16	oligopeptide ABC transporter (oppAIV)	0.2704	0.4055	0.2443	0.3597	1.0765	1	0.9376	0.871	0.7244	0.4169	0.6252	0.3733	0.61±0.29	0.0009
162	BB_0068	conserved hypothetical protein	0.6138	0.4285	0.7112	0.4966	0.7112	0.631	0.8241	0.7311	0.4656	0.5702	0.5395	0.6607	0.62±0.11	0.0001
322	BB_O33	conserved hypothetical protein	0.7112	0.5916	0.8017	0.673	0.5861	0.4246	0.6668	0.4831	0.6918	0.4529	0.787	0.5152	0.62±0.12	0.0001
236	BB_0364	conserved hypothetical protein	0.6918	0.6668	0.7447	0.7244	0.5546	0.4742	0.6026	0.5152	0.5702	0.6427	0.6194	0.6982	0.63±0.08	0.0001
358	BB_B22	conserved hypothetical protein	0.5808	0.5152	0.6486	0.5754	0.6792	0.7447	0.7656	0.8318	0.631	0.4207	0.7047	0.4699	0.63±0.12	0.0001
120	BB_0195	cell division control protein 27 putative	0.6486	0.673	0.631	0.6546	0.631	0.6427	0.6081	0.6252	0.7244	0.6081	0.7047	0.5861	0.64±0.04	0.0001
327	BB_0488	ribosomal protein L14 (rplN)	0.5346	0.7516	0.4786	0.6607	0.7244	0.7178	0.6427	0.6368	0.7798	0.5395	0.6918	0.4786	0.64±0.1	0.0001
166	BB_0153	superoxide dismutase (sodA)	0.5754	0.863	0.5152	0.7727	0.7112	0.6982	0.6486	0.6368	0.631	0.5346	0.5754	0.492	0.64±0.11	0.0001
94	BB_0137	long-chain-fatty-acid CoA ligase	0.7656	0.9462	0.7178	0.879	0.5105	0.597	0.4786	0.5598	0.6486	0.4875	0.6081	0.4529	0.64±0.15	0.0001

149	BB_B28	predicted coding region BBB28	0.5598	0.9376	0.6918	1.1482	0.4055	0.6081	0.4966	0.7379	0.5248	0.4055	0.6427	0.5152	0.64±0.21	0.0001
248	BB_0653	protein-export membrane protein (secF) predicted coding region BB0011	0.6668	0.7112	0.6607	0.7047	0.6026	0.6546	0.597	0.6486	0.5598	0.7047	0.5495	0.6982	0.65±0.05	0.0001
170	BB_K13	conserved hypothetical protein	0.3251	0.3664	0.5012	0.5916	0.7727	0.6855	1.2023	1.0765	0.6918	0.1754	1.0864	0.278	0.65±0.33	0.0041
49	BB_0087	L-lactate dehydrogenase (ldh)	0.5248	0.8551	0.5105	0.7943	0.9462	0.863	0.879	0.7943	0.5297	0.3664	0.5012	0.3404	0.66±0.21	0.0002
121	BB_0203	Lambda CII stability-governing protein (hflK)	0.8166	0.7656	0.6668	0.6252	0.7244	0.7178	0.597	0.5861	0.912	0.4699	0.7447	0.3802	0.67±0.14	0.0001
178	BB_0761	conserved hypothetical protein chemotaxis response regulator (cheY-2)	0.3631	0.7656	0.3342	0.6855	0.7798	0.8091	0.6982	0.7178	0.8241	0.7798	0.7311	0.6918	0.68±0.16	0.0001
285	BB_0227	predicted coding region BB0227	0.7047	0.7656	0.7727	0.8472	0.5297	0.5702	0.5754	0.6026	0.4246	0.871	0.4831	0.9638	0.68±0.16	0.0001
115	BB_0069	aminopeptidase II	0.492	0.863	0.4285	0.7379	0.8318	0.8954	0.7244	0.7656	0.8091	0.4831	0.6982	0.4246	0.68±0.17	0.0001
195	BB_0463	nucleoside-diphosphate kinase (ndk)	0.6982	0.9036	0.6546	0.8472	0.4207	0.6918	0.3945	0.6486	0.5649	0.929	0.5346	0.871	0.68±0.17	0.0001
177	BB_0226	seryl-tRNA synthetase (serS)	0.6368	0.4699	0.863	0.6486	0.4613	0.7311	0.6252	0.9817	0.7586	0.4246	1.0186	0.5754	0.68±0.19	0.0001
106	BB_0151	N-acetylglucosamine-6-phosphate deacetylase (nagA)	0.6668	0.7178	0.6486	0.7047	0.8318	1.0186	0.8166	1.0093	0.7112	0.1803	0.7047	0.182	0.68±0.25	0.0016
163	BB_0537	conserved hypothetical protein	1.1169	1.0186	0.8872	0.8091	0.912	0.597	0.7244	0.4786	0.7516	0.166	0.5808	0.1247	0.68±0.3	0.0044
6	BB_0366	aminopeptidase I (yscI)	0.492	0.6982	0.4207	0.5105	1.2359	1.2134	0.929	0.9036	0.7943	0.1706	0.6081	0.1406	0.68±0.34	0.0095
165	BB_P38	erpA ErpA	0.7178	0.6368	0.6855	0.6081	0.8166	0.7586	0.787	0.7244	0.863	0.4571	0.8241	0.4365	0.69±0.13	0.0001
24	BB_0445	fructose-bisphosphate aldolase (fba)	0.3802	0.8241	0.2582	0.5495	1.3183	1.2246	0.863	0.7943	0.7447	0.5105	0.5012	0.3436	0.69±0.32	0.0088
355	BB_0642	spermidine/putrescine ABC transporter ATP-binding protein (potA)	0.5702	0.7516	0.6982	0.9376	0.9638	0.863	1.2023	1.0666	0.4246	0.177	0.52	0.2208	0.7±0.31	0.0086
161	BB_0335	oligopeptide ABC transporter ATP-binding protein (oppF)	0.8017	0.4831	1.0568	0.6252	0.6427	0.6252	0.8551	0.8166	0.6918	0.4406	0.912	0.5808	0.71±0.17	0.0002
242	BB_0300	cell division protein (ftsA)	0.929	0.879	0.8954	0.8472	0.8472	0.7178	0.8166	0.7047	0.2249	0.7379	0.2188	0.7047	0.71±0.23	0.0016
291	BB_0551	chemotaxis response regulator (cheY-1)	0.7586	0.7586	0.8091	0.8017	0.6368	0.6668	0.6792	0.7047	0.4365	0.9376	0.4571	1	0.72±0.16	0.0001
299	BB_0606	conserved hypothetical protein	0.8954	0.8318	0.7798	0.7244	0.8954	0.7178	0.7798	0.631	0.8551	0.4966	0.7516	0.4365	0.73±0.14	0.0001
138	BB_0034	predicted coding region BB0034	0.8241	0.9727	0.5702	0.6855	0.8954	1.0375	0.6138	0.7178	0.5495	0.8954	0.3873	0.6194	0.73±0.19	0.0006
307	BB_B18	GMP synthase (guaA)	0.5395	0.7943	0.5495	0.8091	0.7516	0.871	0.7656	0.8872	0.9817	0.3837	1	0.3873	0.73±0.2	0.0001
175	BB_0553	predicted coding region BB0553	0.7244	0.879	0.6607	0.8017	0.6546	0.8017	0.6026	0.7244	1.1376	0.3631	1.0375	0.3311	0.73±0.23	0.002
264	BB_L34	conserved hypothetical protein	0.929	0.5495	1.0568	0.6252	0.673	0.5297	0.7727	0.6081	1.0186	0.3873	1.1588	0.4055	0.73±0.25	0.0038
323	BB_0449	conserved hypothetical protein	1.2023	1.0471	0.9376	0.7943	0.7798	0.7447	0.6026	0.5861	0.7656	0.3664	0.5916	0.2911	0.73±0.25	0.004
338	BB_Q34	bdrW BdrW	0.7586	0.6855	0.7112	0.6427	0.863	0.7586	0.8166	0.7112	0.7586	0.7656	0.7178	0.7244	0.74±0.06	0.0001
160	BB_0376	S-adenosylmethionine synthetase (metK)	0.787	0.912	0.6546	0.7516	0.7727	0.8241	0.6368	0.6792	1.0186	0.6026	0.8318	0.5058	0.75±0.14	0.0001
203	BB_0103	predicted coding region BB0103	0.9036	0.7586	1.0186	0.8472	0.6918	0.7943	0.7656	0.9036	0.5346	0.6486	0.5861	0.7047	0.76±0.13	0.0001
348	BB_0546	predicted coding region BB0546	0.8091	0.8241	0.7379	0.7516	1	0.7656	0.9204	0.7047	0.8241	0.5152	0.7586	0.4742	0.76±0.14	0.0001
215	BB_0695	ribosomal protein S16 (rpsP)	0.492	0.3311	0.5445	0.3837	0.6855	0.8166	0.7798	0.9376	0.7943	1.1272	0.912	1.2942	0.76±0.28	0.0149
205	BB_0405	predicted coding region BB0405	0.7178	0.673	0.8091	0.7586	0.9376	1.0375	1.0666	1.1695	0.871	0.0614	0.9817	0.0692	0.76±0.34	0.0423
226	BB_0047	conserved hypothetical protein	1.2823	0.9638	0.8872	0.6668	0.9036	1.2474	0.6486	0.863	0.8551	0.0946	0.597	0.0759	0.76±0.36	0.0476
67	BB_0752	predicted coding region BB0752	0.6194	0.6982	0.7727	0.879	0.6194	0.863	0.787	1.0765	0.6368	0.6855	0.8091	0.8395	0.77±0.13	0.0001
320	BB_0840	predicted coding region BB0840	0.9036	0.9036	0.9817	0.9908	0.6668	0.6486	0.7244	0.7047	0.6138	0.7311	0.6668	0.7943	0.78±0.13	0.0001
122	BB_0325	predicted coding region BB0325	0.7311	0.9376	0.7112	0.879	0.9727	0.8872	0.9376	0.863	0.7943	0.4529	0.7656	0.4246	0.78±0.17	0.0014
125	BB_B17	IMP dehydrogenase (guaB)	1.1482	1.0864	0.7656	0.7447	0.7798	0.8954	0.5152	0.6081	1.3677	0.3499	0.929	0.2228	0.78±0.32	0.0455
156	BB_0061	thioredoxin (trxA)	0.4487	1.1588	0.3048	0.787	1.1803	1.3428	0.8091	0.9204	1.2359	0.169	0.8395	0.1127	0.78±0.41	0.0962
267	BB_0169	translation initiation factor 1 (infA)	1.0375	0.7379	1.0186	0.7178	0.5445	0.9376	0.5248	0.912	1.1588	0.3698	1.1272	0.3597	0.79±0.27	0.0257
14	BB_0348	pyruvate kinase (pyk)	0.492	0.673	0.4325	0.5649	1.4588	1.5276	1.2023	1.2942	0.6081	0.3908	0.52	0.3467	0.79±0.42	0.1331
55	BB_0658	phosphoglycerate mutase (gpmA)	0.7244	0.871	0.413	0.4966	1.406	1.6144	0.8395	0.912	0.5861	0.9908	0.3192	0.5702	0.81±0.37	0.1218
344	BB_0702	lipopolysaccharide biosynthesis-related protein (kdtB)	0.6427	0.7516	0.8318	0.9638	1.3932	0.8017	1.803	1.028	0.6486	0.0121	0.8318	0.0128	0.81±0.48	0.2133
213	BB_0705	ribonuclease III (rnc)	0.8318	0.863	0.8091	0.8395	0.8318	0.7379	0.8091	0.7112	0.8954	0.863	0.863	0.8318	0.82±0.05	0.0001
259	BB_0575	CTP synthase (pyrG)	0.8241	0.8241	0.7727	0.7727	0.8551	0.8166	0.8017	0.7656	0.8318	0.955	0.7798	0.8954	0.82±0.05	0.0001
275	BB_0194	conserved hypothetical protein	1.2246	1.1169	0.9036	0.8166	0.673	0.871	0.5012	0.6427	0.6792	1.0864	0.5152	0.7943	0.82±0.22	0.0211
238	BB_0599	cysteinyl-tRNA synthetase (cysS)	0.7798	0.8872	0.7379	0.8395	0.863	0.8954	0.8166	0.8395	0.912	0.7798	0.863	0.7379	0.83±0.06	0.0001
313	BB_F20	conserved hypothetical protein	0.7586	0.7516	0.8091	0.7943	0.9638	0.9036	1.028	0.955	0.871	0.5861	0.9204	0.6252	0.83±0.13	0.0012
362	BB_A59	lipoprotein	0.7047	0.4699	0.9908	0.673	0.52	0.8166	0.6855	1.1588	0.4571	1.1912	0.6486	1.6749	0.83±0.35	0.137
245	BB_0831	xylose operon regulatory protein (xylR-2)	0.8872	0.9204	0.8551	0.8872	0.8954	0.9727	0.863	0.9376	0.5916	0.871	0.5702	0.8395	0.84±0.12	0.0012
287	BB_0207	UTP--glucose-1-phosphate uridylyltransferase (gtaB)	1.028	1.0666	0.9462	0.9817	0.8166	0.6486	0.7516	0.597	0.8017	0.863	0.7447	0.8017	0.84±0.14	0.0027
109	BB_0785	stage V sporulation protein G	1.3932	0.8091	1.1066	0.6486	0.6252	0.6855	0.4742	0.5445	1.0568	1.0186	0.8318	0.8472	0.84±0.26	0.0579
51	BB_0800	N-utilization substance protein A (nusA)	0.879	0.9908	0.673	0.787	0.929	1.0864	0.7447	0.871	1.4859	0.2559	1.1376	0.2014	0.84±0.34	0.1399
150	BB_H37	predicted coding region BBH37	0.7447	1.2246	0.7586	1.2134	0.3133	0.8954	0.3281	0.8954	1.7701	0.0929	1.7378	0.0904	0.84±0.55	0.3523

318	BB_0298	conserved hypothetical protein	0.8472	0.871	0.863	0.879	0.8472	0.787	0.863	0.7943	0.8241	0.863	0.8318	0.871	0.85±0.03	0.0001
357	BB_0107	N-utilization substance protein B (nusB)	0.8872	0.863	0.9462	0.929	0.8551	0.787	0.912	0.8395	0.7516	0.8166	0.8017	0.871	0.85±0.06	0.0001
265	BB_0327	glycerol-3-phosphate O-acyltransferase putative	0.9727	1.1376	0.8472	1	0.9462	0.8472	0.8318	0.7516	0.8551	0.6918	0.7516	0.6138	0.85±0.14	0.005
146	BB_0144	glycine betaine L-proline ABC transporter (proX)	0.912	0.9727	1.0965	1.1803	0.5546	0.912	0.6792	1.1169	0.5546	0.6918	0.673	0.863	0.85±0.21	0.037
305	BB_0582	carboxypeptidase putative	0.9727	0.912	0.871	0.8091	0.8872	1.2246	0.787	1.0965	1.406	0.0114	1.2474	0.0113	0.85±0.42	0.2687
220	BB_0596	methyl-accepting chemotaxis protein (mcp-2)	0.9036	0.929	0.9036	0.929	0.7943	0.787	0.7943	0.787	0.8551	0.912	0.8551	0.912	0.86±0.06	0.0001
270	BB_0597	methyl-accepting chemotaxis protein (mcp-3)	1.0471	0.9727	0.9727	0.8954	0.8472	0.8166	0.7798	0.7516	0.8472	0.8091	0.7798	0.7447	0.86±0.09	0.0003
21	BB_0127	ribosomal protein S1 (rpsA)	1.1169	1.2474	0.7943	0.8954	0.8395	1.1482	0.5916	0.8166	1.0093	0.6486	0.7047	0.4487	0.86±0.23	0.0614
262	BB_0146	glycine betaine L-proline ABC transporter ATP-binding protein (proV)	1.2706	1.0765	1.2359	1.0375	0.8954	1.0765	0.863	1.0471	0.929	0.0113	0.8954	0.0112	0.86±0.4	0.2781
258	BB_0196	peptide chain release factor 1 (prfA)	0.787	0.912	0.7112	0.8241	0.912	0.9036	0.8241	0.8166	0.9727	1.0093	0.879	0.912	0.87±0.08	0.0002
386	BB_R34	conserved hypothetical protein	1	0.8954	1	0.8954	0.8551	0.8318	0.8551	0.8318	0.6918	0.9638	0.6918	0.9638	0.87±0.1	0.0014
189	BB_0126	predicted coding region BB0126	0.879	1.0666	1.1066	1.3428	0.4966	0.7244	0.6252	0.912	0.5754	0.863	0.7112	1.1066	0.87±0.24	0.0978
271	BB_0561	phosphogluconate dehydrogenase decarboxylating (gnd)	0.9376	1.2359	0.863	1.1272	1.0965	0.9462	1	0.863	1.0965	0.1191	1	0.1096	0.87±0.35	0.2338
254	BB_0239	deoxyguanosine/deoxyadenosine kinase(I) subunit 2 (dck)	0.8551	0.9638	0.871	0.9817	0.863	0.8551	0.879	0.871	0.863	0.8318	0.879	0.8472	0.88±0.04	0.0001
243	BB_0074	peptide chain release factor 2 (prfB)	0.929	0.8166	1.0765	0.9462	0.7943	0.7656	0.9204	0.8872	0.8091	0.7656	0.9376	0.8872	0.88±0.09	0.0008
266	BB_Q40	plasmid partition protein putative	1.0765	1.0186	1.5136	1.4454	0.673	0.7047	0.955	0.9908	0.9036	0.0223	1.2823	0.0268	0.88±0.46	0.4184
182	BB_G01	predicted coding region BBG01	0.1432	0.0457	0.1271	0.0406	1.6904	1.4997	1.4723	1.2942	2.0137	0.2606	1.7378	0.2399	0.88±0.76	0.6111
276	BB_0657	ribose 5-phosphate isomerase (rpi)	0.955	0.871	1.028	0.929	0.7798	0.8472	0.8318	0.9036	0.7516	0.9727	0.8091	1.0375	0.89±0.09	0.0023
370	BB_0324	predicted coding region BB0324	0.9036	1.2823	0.6855	0.9727	0.879	0.955	0.6668	0.7244	1.0864	1	0.8241	0.7586	0.89±0.17	0.068
279	BB_0801	translation initiation factor 2 (infB)	1.9055	0.8551	1.7061	0.7798	1.1169	0.5649	1.0186	0.4966	0.2655	0.879	0.2399	0.8091	0.89±0.49	0.4564
169	BB_0757	ATP-dependent Clp protease proteolytic component (clpP-2)	0.9376	0.9462	1.0186	1.0375	0.7656	0.879	0.8318	0.9638	0.9376	0.7178	1.028	0.7798	0.9±0.1	0.0104
410	BB_0646	predicted coding region BB0646	0.8318	0.7727	0.787	0.7311	0.9908	0.9908	0.9376	0.9376	0.8872	1.1066	0.8395	1.0471	0.9±0.11	0.0165
361	BB_0027	predicted coding region BB0027	0.9036	0.8395	0.9817	0.9036	0.7244	0.912	0.787	0.9908	0.6138	1.2023	0.6607	1.3062	0.9±0.2	0.1248
204	BB_0176	methanol dehydrogenase regulator (moxR)	0.7943	0.7798	1.1066	1.0666	0.7798	1.0471	1.0666	1.4322	0.6607	0.4742	0.8954	0.6546	0.9±0.25	0.1948
183	BB_0141	membrane fusion protein (mtrC)	0.912	0.8872	0.912	0.8872	0.9462	0.879	0.955	0.879	0.8954	0.9638	0.8954	0.9638	0.91±0.03	0.0001
316	BB_0319	exported protein (tpn38b)	0.8872	0.8395	0.929	0.879	0.929	0.8472	0.9727	0.8872	0.9462	0.863	0.9908	0.9036	0.91±0.05	0.0001
345	BB_0644	conserved hypothetical protein	0.9204	1.0186	0.9376	1.028	0.9638	0.871	0.9727	0.8872	0.8091	0.8395	0.8241	0.8472	0.91±0.07	0.0016
223	BB_0005	tryptophanyl-tRNA synthetase (trsA)	0.8551	0.8091	1.0471	1	0.8551	0.787	1.0568	0.9727	0.7798	0.787	0.9638	0.9638	0.91±0.1	0.0104
346	BB_0568	protein-glutamate methylesterase (cheB-2)	0.8166	0.863	0.9908	1.0375	0.879	0.7943	1.0666	0.9638	0.8241	0.7656	1	0.929	0.91±0.1	0.0109
186	BB_0032	predicted coding region BB0032	0.871	1.1482	0.7727	1.0375	1.0375	1.0568	0.9376	0.955	0.9204	0.7379	0.8318	0.673	0.91±0.14	0.0642
221	BB_0543	predicted coding region BB0543	0.787	1.0093	0.6918	0.8872	0.7656	1.1912	0.6982	1.0471	1.4322	0.6368	1.2589	0.5445	0.91±0.26	0.2958
126	BB_0112	ribosomal protein L9 (rplI)	0.8395	0.9908	0.7244	0.8551	1.0965	1.1695	0.9462	1.0093	1.3932	0.3767	1.1912	0.2992	0.91±0.31	0.34
71	BB_0627	vacuolar X-prolyl dipeptidyl aminopeptidase I (pepX)	0.7727	0.7112	0.8954	0.8091	1.3804	1.1588	1.5996	1.3677	0.413	0.597	0.4831	0.7047	0.91±0.37	0.4207
273	BB_0118	zinc protease putative	0.9036	0.871	0.9727	0.9376	0.863	0.8872	0.929	0.955	0.9036	0.9036	0.9727	0.9727	0.92±0.04	0.0001
360	BB_0676	phosphoglycolate phosphatase (gph)	0.8872	0.9727	0.9204	1.0186	0.9376	0.8551	0.9817	0.8954	0.9204	0.8551	0.955	0.8872	0.92±0.05	0.0003
200	BB_0512	predicted coding region BB0512	0.7943	1	0.879	1.0965	0.8241	0.8551	0.912	0.9462	0.929	0.8318	1.0186	0.9204	0.92±0.08	0.008
293	BB_0621	4-methyl-5(b-hydroxyethyl)-thiazole monophosphate biosynth protein (thiJ)	0.879	0.9376	1.0765	1.1482	0.8017	0.7244	0.9817	0.8872	0.8395	0.7798	1.0375	0.955	0.92±0.12	0.053
315	BB_0222	glucose-6-phosphate 1-dehydrogenase putative	0.8872	0.7447	1.1803	0.9908	0.9204	0.7727	1.2246	1.028	0.673	0.7244	0.8954	0.9638	0.92±0.17	0.1268
137	BB_0121	ribosome releasing factor (frr)	0.6546	0.8318	0.7244	0.9462	1.0568	1.0093	1.2023	1.1376	0.9817	0.6486	1.1066	0.7311	0.92±0.19	0.1795
301	BB_0261	predicted coding region BB0261	0.8551	0.912	0.8954	0.955	1.0375	0.8472	1.0864	0.8872	0.929	0.8472	0.9727	0.8872	0.93±0.07	0.006
364	BB_0152	glucosamine-6-phosphate isomerase (nagB)	0.8472	0.9376	0.7943	0.879	1.1482	1.1169	1.0765	1.0375	0.9204	0.8017	0.863	0.7447	0.93±0.13	0.1013
235	BB_0629	PTS system fructose-specific IIABC component (fruA-2)	1	0.879	1.0568	0.9376	0.7379	1.1272	0.7798	1.2023	0.7178	0.955	0.7656	1.0186	0.93±0.15	0.1625
369	BB_R32	conserved hypothetical protein	1.1066	0.9036	1.3305	1.0864	0.8017	0.9376	0.9638	1.1376	0.6982	0.6081	0.8395	0.7379	0.93±0.2	0.2657
86	BB_0754	ABC transporter ATP-binding protein	0.929	1.2474	1	1.406	0.8017	1.0186	0.8954	1.1169	0.7311	0.5754	0.8017	0.6546	0.93±0.23	0.3499
92	BB_0039	predicted coding region BB0039	0.9462	1.1066	1.2134	1.4454	0.5916	0.5916	0.7727	0.7656	0.8872	0.7047	1.1482	0.955	0.93±0.25	0.3586
241	BB_0135	histidyl-tRNA synthetase (hisS)	1.2474	0.955	1.5704	1.2134	0.7379	0.5916	0.929	0.7244	0.7727	0.6194	0.9727	0.7798	0.93±0.28	0.3979
363	BB_0755	conserved hypothetical protein	1.0568	0.8872	1.0666	0.8954	1.0568	0.8872	1.0568	0.8954	0.879	0.871	0.879	0.879	0.94±0.08	0.0419
104	BB_0299	cell division protein (ftsZ)	1.1588	0.9817	1.2023	1.028	1.1912	1.028	1.2246	1.0666	0.9376	0.2333	0.9727	0.2606	0.94±0.32	0.554
12	BB_0518	heat shock protein 70 (dnaK-2)	0.9727	1.3183	0.6668	0.9204	0.7656	1.0864	0.5395	0.7516	1.7378	0.7727	1.1695	0.5248	0.94±0.34	0.5376
136	BB_0064	methionyl-tRNA formyltransferase (fmt)	0.955	0.871	1.5704	1.406	0.7727	0.7943	1.2823	1.2706	0.3404	0.5248	0.5445	0.9817	0.94±0.36	0.6121
330	BB_G09	conserved hypothetical protein	0.1047	0.3532	0.0679	0.2249	2.0324	1.4723	1.2706	0.912	1.4588	1.5136	0.929	0.9462	0.94±0.61	0.7531
232	BB_0065	polypeptide deformylase (def)	0.9817	0.955	0.9908	0.9638	0.9908	0.863	1	0.871	0.929	0.929	0.9376	0.9376	0.95±0.04	0.0014

164	BB_A76	thymidylate synthase-complementing protein (thy1)	0.9908	0.863	1.0666	0.9376	0.9204	0.8551	0.9908	0.9204	0.955	0.879	1.0375	0.9462	0.95±0.06	0.0177
281	BB_0182	flagellar hook-associated protein 3 (flgL)	0.9036	0.7727	1.028	0.879	0.863	0.929	0.9817	1.0568	0.9638	0.9036	1.0965	1.028	0.95±0.09	0.092
198	BB_0281	flagellar motor rotation protein A (motA)	0.8166	0.8472	0.9638	1	0.9204	0.8551	1.0864	1.0093	0.8017	1	0.9462	1.1803	0.95±0.11	0.1713
111	BB_0368	glycerol-3-phosphate dehydrogenase NAD(P)+ (gpsA)	0.9817	1.1376	0.9908	1.1482	1.0471	1.0186	1.0568	1.028	0.8318	0.6792	0.8472	0.6918	0.95±0.15	0.3427
114	BB_0283	flagellar hook protein (flgE)	0.673	0.5808	0.8318	0.7244	1.0471	1.0765	1.3183	1.3552	0.912	0.7656	1.1482	0.9376	0.95±0.24	0.4795
208	BB_C03	conserved hypothetical protein	0.0322	0.0425	0.0296	0.0457	1.0965	1.7061	1.0965	1.7061	1.7219	1.0965	1.7219	1.0965	0.95±0.69	0.8127
70	BB_0789	cell division protein (ftsH)	0.9376	1.0965	1	1.1695	0.9462	0.879	1	0.9376	0.8551	0.871	0.912	0.9204	0.96±0.09	0.1706
329	BB_0279	flagellar protein (fliL)	1.0765	0.9376	1.028	0.8954	0.9727	0.9908	0.9376	0.955	1.0864	0.8091	1.0375	0.7798	0.96±0.09	0.1665
218	BB_0669	chemotaxis histidine kinase (cheA-2)	1.1169	1.0375	1.0093	0.929	1.028	0.8395	0.929	0.7586	0.9727	1.0864	0.871	0.9817	0.96±0.1	0.2473
225	BB_0664	predicted coding region BB0664	0.9908	1.1272	0.8166	0.929	1.0471	1.0864	0.863	0.8872	1.028	1.0375	0.8472	0.8551	0.96±0.1	0.2125
288	BB_0630	1-phosphofructokinase (fruK)	1.2942	0.787	1.5849	0.955	0.7244	1.2589	0.879	1.5417	1.0666	0.0692	1.3062	0.0738	0.96±0.48	0.7952
95	BB_0493	ribosomal protein L6 (rplF)	0.9462	0.9817	0.9638	1	0.9462	0.9462	0.955	0.9638	0.9727	1	0.9908	1.0093	0.97±0.02	0.0018
302	BB_0486	ribosomal protein L29 (rpmC)	1	0.9817	1.028	1.0093	0.9462	0.9462	0.9727	0.9727	0.863	1.0093	0.8872	1.0375	0.97±0.05	0.0886
314	BB_0544	phosphoribosyl pyrophosphate synthetase (prs)	1.028	0.9204	1.0965	0.9817	0.955	0.9204	1.0186	0.9817	0.9036	0.8954	0.955	0.9462	0.97±0.06	0.0761
240	BB_0671	chemotaxis operon protein (cheX)	0.9204	1.0186	0.8241	0.912	0.955	1.0568	0.863	0.955	1.0765	1.0965	0.9638	0.9817	0.97±0.08	0.2187
280	BB_0437	chromosomal replication initiator protein (dnaA)	0.9727	0.879	1.0186	0.9204	0.7943	0.9727	0.8318	1.0186	1	1.0765	1.0471	1.1272	0.97±0.1	0.3424
331	BB_K24	conserved hypothetical protein	0.929	0.955	0.9204	0.9462	0.8241	0.863	0.8166	0.8551	1.0965	1.1695	1.0864	1.1588	0.97±0.12	0.4094
303	BB_0704	acyl carrier protein	0.7586	1	0.8166	1.0765	0.7516	1.0765	0.8091	1.1588	0.955	1.0471	1.0375	1.1376	0.97±0.14	0.4787
155	BB_0394	transcription antitermination factor (nusG)	1.1169	1.2023	1.0864	1.1695	0.912	0.9204	0.8872	0.8954	0.7798	1.0093	0.7586	0.9727	0.98±0.14	0.5742
326	BB_0181	flagellar hook-associated protein (flgK)	1.0186	0.8472	1.0864	0.9036	1.1912	1.0093	1.2706	1.0864	0.8954	0.7178	0.955	0.7586	0.98±0.16	0.66
81	BB_0594	arginyl-tRNA synthetase (argS)	1.2134	0.9727	1.6144	1.2823	0.7516	0.929	0.9727	1.2134	0.5546	0.6486	0.7244	0.8551	0.98±0.29	0.8051
110	BB_0715	rod shape-determining protein (mreB-1)	0.9727	0.9817	0.9817	0.9908	0.9908	0.9908	1	1	1	0.9908	1.0186	1	0.99±0.01	0.0703
230	BB_H32	antigen P35 putative	0.9462	0.9817	0.9638	1	1.0186	1	1.0375	1.028	0.9908	0.955	1.0186	0.9817	0.99±0.03	0.4558
269	BB_0694	signal recognition particle protein (ffh)	1.0765	1.1695	0.9727	1.0666	1.0093	0.9638	0.9204	0.871	1.0186	1.0093	0.929	0.9204	0.99±0.08	0.8042
222	BB_0482	ribosomal protein S19 (rpsS)	1.0471	1.4723	1.0965	1.5417	0.912	0.9817	0.955	1.028	1.0568	0.3373	1.1066	0.3404	0.99±0.34	0.9222
54	BB_0639	spermid/putresc ABC transporter (potD)	0.9908	1.2359	0.7244	0.8872	1.3305	1.4588	0.9727	1.0666	1.4454	0.4285	1.0666	0.3192	0.99±0.35	0.9547
172	BB_0286	flagellar protein (flbB)	0.9727	0.9908	0.955	0.9817	1.0471	1	1.028	0.9908	1.0375	1	1.028	0.9817	1±0.03	0.8944
227	BB_0735	rare lipoprotein A (rlpA)	0.955	1	0.9376	0.9817	1.0093	1.0568	0.9908	1.0375	1.0666	0.9817	1.0471	0.9638	1±0.04	0.851
192	BB_0557	phosphocarrier protein HPr (ptsH-2)	0.9727	0.9376	1.0864	1.0471	0.9036	0.9462	1.0186	1.0568	1.0186	0.8954	1.1376	1	1±0.07	0.9374
294	BB_0806	predicted coding region BB0806	0.9638	0.9376	1.1066	1.0666	0.929	0.955	1.0666	1.0965	0.8954	0.9462	1.0186	1.0765	1±0.07	0.8258
351	BB_K50	immunogenic protein P37	0.879	0.9727	0.9036	1.0093	0.9376	1.028	0.9638	1.0568	1.0765	1.0093	1.1169	1.0375	1±0.07	0.9713
286	BB_0200	D-alanine--D-alanine ligase (ddlA)	1.7378	0.871	1.7219	0.8551	1.2942	1.1803	1.2823	1.1588	0.9036	0.0506	0.8872	0.0545	1±0.51	0.9989
306	BB_A60	surface lipoprotein P27	0.3981	0.0731	0.4613	0.0904	0.8954	1.1482	1.0568	1.3428	1.556	1.4588	1.8197	1.7061	1±0.59	0.9976
349	BB_0650	predicted coding region BB0650	0.9817	1.0375	0.9727	1.028	1	0.9817	0.9908	0.9727	1	1.0864	0.9908	1.0765	1.01±0.04	0.3971
187	BB_0277	flagellar motor switch protein (fliN)	1.0093	1.0471	0.9376	0.9727	0.9727	1.0864	0.9036	1.0093	1.0375	1.1169	0.9638	1.0375	1.01±0.06	0.6662
209	BB_0402	prolyl-tRNA synthetase (proS)	1.0568	1.0093	1.0864	1.0471	1.0666	0.9908	1.0965	1.0186	0.9036	0.9462	0.929	0.9817	1.01±0.06	0.5541
201	BB_0125	predicted coding region BB0125	0.8241	0.929	0.9462	1.0765	0.871	1.0965	1	1.2589	1.3932	0.5297	1.6144	0.6138	1.01±0.29	0.8881
268	BB_0025	conserved hypothetical protein	1.0093	1.1066	0.929	1.0186	1.0965	1.0965	1.0093	1.0093	1.0568	1.0375	0.9727	0.955	1.02±0.05	0.1603
272	BB_K19	predicted coding region BBK19	1.0666	1.0765	1.028	1.0471	1.0471	1.1482	1.0186	1.1169	1.0093	0.8872	0.9817	0.863	1.02±0.08	0.3363
234	BB_0820	predicted coding region BB0820	1.2474	1.1272	1.3552	1.2246	0.879	0.8872	0.9462	0.9638	1.1169	0.6252	1.2134	0.6792	1.02±0.22	0.7455
69	BB_0786	general stress protein (ctc)	1.1912	1.1695	0.9908	1.0186	0.9462	1.3305	0.8318	1.1482	1.3183	0.597	1.1482	0.5346	1.02±0.25	0.8052
26	BB_0104	periplasmic serine protease DO (htrA)	1.3428	1.3932	0.929	0.9817	1.3677	1.3804	0.9204	0.9462	0.9204	0.8395	0.6368	0.5808	1.02±0.27	0.8141
173	BB_0477	ribosomal protein S10 (rpsJ)	1.028	1.0568	1.0375	1.0666	1	1.0186	1.0093	1.028	1.0375	0.9817	1.0471	0.9908	1.03±0.03	0.0067
129	BB_0290	flagellar motor switch protein (fliG-2)	0.929	1.0186	0.955	1.0375	1	1.0375	1.028	1.0666	1.0765	1.0471	1.0965	1.0666	1.03±0.05	0.0586
142	BB_0270	flagellar-associated GTP-binding protein (flhF)	1.0471	1.028	1.0965	1.0765	0.9908	0.9727	1.0375	1.0186	0.9376	1.028	0.9817	1.0864	1.03±0.05	0.0991
190	BB_0133	predicted coding region BB0133	1.028	1.0186	1.1066	1.0965	1.0093	1	1.0864	1.0765	0.929	0.955	1	1.028	1.03±0.05	0.1098
333	BB_0159	predicted coding region BB0159	1.0375	1.0864	1.1066	1.1588	0.912	0.9817	0.9727	1.0471	0.9908	1	1.0568	1.0666	1.03±0.06	0.101
197	BB_0063	predicted coding region BB0063	1.1482	1.0864	1.0471	0.9908	1.1272	1.0965	1.028	1.0093	1.0375	0.9817	0.9462	0.9036	1.03±0.07	0.1395
359	BB_0819	cytidylate kinase (cmk-2)	0.8954	1.0965	0.8395	1.028	1.0765	1.0186	1.0093	0.955	1.1066	1.1588	1.0471	1.0864	1.03±0.09	0.3399
249	BB_0278	flagellar motor switch protein (fliM)	1.0093	0.9376	1.0765	1.0093	1.0765	1	1.1482	1.0765	0.9817	1.0471	1.0471	1.1169	1.04±0.06	0.0267
179	BB_0371	glycyl-tRNA synthetase (glyS)	0.9908	1	1.0666	1.0765	1.0375	1.0093	1.1169	1.0864	1	1.0186	1.0765	1.0864	1.05±0.04	0.0028
154	BB_0601	serine hydroxymethyltransferase (glyA)	1.0864	1.1482	0.9908	1.0471	1.0965	1.0471	1	0.955	1.1482	1.0568	1.0471	0.9638	1.05±0.06	0.023

210	BB_0235	conserved hypothetical protein	1.1588	1.0093	1.2134	1.0666	1.2134	1.0093	1.2706	1.0568	0.8551	0.871	0.8954	0.9204	1.05±0.14	0.302
176	BB_0372	glutamyl-tRNA synthetase (gltX)	1.0568	1.0375	1.1169	1.0965	1.0375	1.0093	1.1066	1.0666	1	1.0093	1.0568	1.0666	1.06±0.04	0.0004
194	BB_0778	ribosomal protein L21 (rplU)	1.028	1.0765	1.0666	1.1272	1.028	1.0666	1.0666	1.1066	1.0666	0.9638	1.1066	1.0093	1.06±0.04	0.0009
300	BB_0458	predicted coding region BB0458	0.9462	0.9908	1.0666	1.1066	1.0471	0.955	1.1695	1.0765	0.8954	1.1588	1.0093	1.2942	1.06±0.11	0.0917
36	BB_0359	carboxyl-terminal protease (ctp)	1.1803	1.3932	1.1803	1.3804	1.028	1.0471	1.028	1.0471	0.7516	0.9908	0.7447	0.9908	1.06±0.19	0.2993
38	BB_0610	trigger factor (tig)	1.1066	1.6749	0.9817	1.5136	1.1169	1.2023	0.9908	1.0765	0.9376	0.6792	0.8395	0.6081	1.06±0.29	0.5073
290	BB_0672	chemotaxis response regulator (cheY-3)	1.1912	1.3804	0.7943	0.9204	1.5136	1.2134	1.0093	0.8017	1.4723	0.8166	0.9817	0.5702	1.06±0.29	0.534
145	BB_0291	flagellar basal-body rod protein (fliF)	0.5808	0.7586	0.6855	0.871	0.8166	1.1803	0.9204	1.4588	1.0666	1.4322	1.2706	1.7219	1.06±0.34	0.5451
298	BB_0228	conserved hypothetical protein	1.6444	0.9204	1.8707	1.0471	1.1803	1.0093	1.3552	1.1482	1.0765	0.1169	1.2246	0.1271	1.06±0.49	0.694
311	BB_J16	conserved hypothetical protein	1.0765	1.1272	1.0765	1.1272	1.1066	1.0666	1.1066	1.0666	1.0666	0.955	1.0666	0.955	1.07±0.05	0.0019
66	BB_0055	triosephosphate isomerase	1.0965	1.4997	0.912	1.2942	1.1482	1.0864	0.955	0.8954	0.8241	1.3183	0.7047	1.1066	1.07±0.22	0.308
131	BB_0253	ATP-dependent protease LA (lon-1)	1.1272	0.6607	1.8365	1.1482	0.8318	0.9462	1.4322	1.5417	0.3436	0.879	0.597	1.5136	1.07±0.43	0.5888
101	BB_0559	PTS system glucose-specific IIA component (crr)	1.1588	1.6293	1.2706	1.7539	1.2589	1.0864	1.3677	1.1912	0.6982	0.3162	0.7586	0.3499	1.07±0.44	0.6058
127	BB_A62	lipoprotein	0.2489	0.2109	0.3532	0.2512	1.406	1.4859	1.8197	1.9231	1.4859	0.7447	1.9409	0.9462	1.07±0.66	0.7388
1	BB_A15	outer surface protein A (ospA)	1.0093	1.0186	1.0864	1.1066	1.0666	1.0568	1.1482	1.1482	1.0568	1.028	1.1376	1.1066	1.08±0.05	0.0001
317	BB_0768	pyridoxal kinase (pdxK)	1.2023	1.1912	1.0666	1.0568	1.2246	1.0375	1.0864	0.929	1.2023	1.0093	1.0666	0.8954	1.08±0.1	0.025
219	BB_0587	methionyl-tRNA synthetase (metG)	1.2823	1.3062	0.863	0.871	1.2706	1.2706	0.8472	0.8472	1.3428	1.3062	0.9036	0.879	1.08±0.22	0.2299
356	BB_0611	ATP-dependent Clp protease proteolytic component (clpP-1)	1.3305	1.3552	1.4191	1.4588	0.871	0.9908	0.9376	1.0666	0.5346	1.1482	0.5754	1.2359	1.08±0.3	0.4065
217	BB_0131	recA protein (recA)	0.9817	1	0.9817	1	1.0666	1.0666	1.0666	1.0666	1.1803	1.2246	1.1803	1.2134	1.09±0.09	0.0076
335	BB_0542	predicted coding region BB0542	1.2706	1.2706	1.0864	1.0864	1.0864	1.2589	0.9204	1.0765	1.1169	1.1376	0.9462	0.9638	1.1±0.12	0.014
151	BB_0248	oligoendopeptidase F (pepF)	1.1169	1.1912	0.9727	1.0471	1.2474	1.1066	1.0965	0.9727	0.912	1.4588	0.8017	1.2823	1.1±0.17	0.0776
253	BB_Q41	conserved hypothetical protein	1.2246	1.1272	1.028	0.9462	1.0093	1.0864	0.8472	0.912	1.1482	1.556	0.9638	1.3062	1.1±0.19	0.1175
144	BB_0101	asparaginyl-tRNA synthetase (asnS)	1.1803	1.0186	1.2023	1.0375	1.2023	1.1169	1.2134	1.1376	1.1066	0.955	1.1272	0.9638	1.11±0.09	0.0022
324	BB_0397	predicted coding region BB0397	1	1.0965	1.1376	1.2474	0.955	1.0568	1.0864	1.2023	0.929	1.1588	1.0666	1.3305	1.11±0.11	0.0101
246	BB_0480	ribosomal protein L23 (rplW)	1.0568	1.2359	0.7798	0.9036	1.4723	1.3062	1.0666	0.9727	1.1272	1.5136	0.8241	1.0965	1.11±0.22	0.1237
228	BB_0613	ATP-dependent protease LA (lon-2)	1.1482	1.0864	1.2246	1.1695	1.0186	1.0093	1.0864	1.0765	1.1588	1.0765	1.2359	1.1482	1.12±0.07	0.0001
45	BB_0751	predicted coding region BB0751	0.8472	1.3183	1.2134	1.8707	1.1482	1.0864	1.6293	1.4997	0.879	0.3105	1.2134	0.4246	1.12±0.44	0.3823
256	BB_0229	ribosomal protein L31 (rpmE)	1.1803	1.0471	1.2246	1.0965	1.0965	1.0965	1.1482	1.1482	1.2246	1.0186	1.2706	1.0568	1.13±0.08	0.0001
341	BB_K17	adenine deaminase (adeC)	1.2359	1.3932	1.2706	1.4191	1.0666	0.9638	1.0864	0.9817	1.0765	0.9727	1.0965	0.9908	1.13±0.15	0.0181
237	BB_0777	adenine phosphoribosyltransferase (apt)	1	0.9376	1.4322	1.3428	1.1803	0.9908	1.6904	1.4191	0.7727	0.7047	1.1066	0.9817	1.13±0.28	0.1522
112	BB_0289	flagellar assembly protein (fliH)	1.1482	0.9817	1.0666	0.9204	1.2589	1.3932	1.1695	1.2823	1.6749	0.6138	1.5276	0.5546	1.13±0.32	0.1973
474	BB_S36	conserved hypothetical protein	1.977	1.406	1.8707	1.3183	2.3121	1.2359	2.1677	1.1588	0.0433	0.0116	0.0417	0.0115	1.13±0.85	0.6244
321	BB_0485	ribosomal protein L16 (rplP)	1.1803	1.0471	1.0965	0.9817	1.2706	1.1169	1.1912	1.0471	1.1912	1.2359	1.1169	1.1482	1.14±0.08	0.0002
251	BB_0837	excinuclease ABC subunit A (uvrA)	1.2823	0.9817	1.3932	1.0666	0.9908	0.9817	1.0666	1.0666	1.1695	1.1376	1.2706	1.2359	1.14±0.13	0.0048
274	BB_0693	xylose operon regulatory protein (xylR-1)	1.1272	1.1803	1.1482	1.2134	0.9462	1.1169	0.9727	1.1482	1.0093	1.3804	1.028	1.4191	1.14±0.14	0.0069
231	BB_0429	predicted coding region BB0429	0.9036	1.5136	0.7112	1.1695	1.5417	1.7378	1.1912	1.3552	1.6749	0.3499	1.2942	0.2655	1.14±0.47	0.3363
41	BB_0108	basic membrane protein	1.0666	1.3932	1.1695	1.4997	0.8954	1.1376	0.9817	1.2359	1.3062	0.8017	1.406	0.863	1.15±0.22	0.0499
61	BB_I36	antigen P35 putative	1.0375	0.7943	0.9817	0.7586	1.3183	1.3677	1.2589	1.2942	1.8365	0.7112	1.7539	0.673	1.15±0.37	0.2146
334	BB_0720	threonyl-tRNA synthetase (thrZ)	0.8241	1.3305	1.2942	2.0512	0.6546	0.7943	1.0186	1.2359	0.879	0.912	1.3804	1.406	1.15±0.37	0.207
337	BB_0804	ribosomal protein S15 (rpsO)	1.3062	1.406	1.0864	1.1695	1.2359	1.1803	1.0375	0.9908	1.2023	1.2589	1.0093	1.0471	1.16±0.12	0.0012
283	BB_0738	valyl-tRNA synthetase (valS)	1.3932	1.3183	1.3305	1.2589	1.0093	1.1169	0.955	1.0666	1.1272	1.1803	1.0765	1.1169	1.16±0.13	0.0017
105	BB_0795	outer membrane protein	0.9908	1.0666	1.5276	1.6293	1.0093	0.9817	1.5417	1.4997	0.8551	0.6427	1.3552	0.9727	1.17±0.31	0.0901
284	BB_0342	glu-tRNA amidotransferase subunit A (gluA)	1.1169	1.1169	1.2474	1.2474	1.2246	1.1482	1.3677	1.2823	1.1803	0.912	1.3062	1.0186	1.18±0.12	0.0005
289	BB_0725	conserved hypothetical protein	1.4191	1.2589	1.4191	1.2589	1.0965	1.2589	1.0965	1.2589	1.2942	0.7379	1.2942	0.7379	1.18±0.22	0.0206
354	BB_K07	predicted coding region BBK07	1.7378	1.8707	1	1.0765	1.5704	1.2246	0.9036	0.7047	1.1912	1.3552	0.6855	0.7798	1.18±0.38	0.1539
133	BB_P33	conserved hypothetical protein	1.8197	1.8365	1.7061	1.7219	0.2729	0.7798	0.2606	0.7311	1.9409	0.7047	1.8197	0.6138	1.18±0.64	0.3637
309	BB_0288	flagellum-specific ATP synthase (fliI)	1.1588	1.2589	1.1482	1.2359	1.2474	1.1482	1.2359	1.1376	1.0375	1.3552	1.028	1.3305	1.19±0.1	0.0001
308	BB_0387	ribosomal protein S12 (rpsL)	1.1066	1.1376	0.929	0.955	1.4322	1.3062	1.2023	1.0965	1.4322	1.3428	1.2023	1.1169	1.19±0.16	0.0023
130	BB_0391	ribosomal protein L10 (rplJ)	1.4997	1.5417	1.3428	1.3552	1.1695	1.2942	1.028	1.1482	0.9638	1.0965	0.863	0.9638	1.19±0.21	0.0123
97	BB_0739	predicted coding region BB0739	1.0965	1.3305	1.1912	1.406	1.2474	1.4322	1.3428	1.5276	1.0765	0.7047	1.1803	0.7656	1.19±0.24	0.0235
88	BB_0407	mannose-6-phosphate isomerase (manA)	1.1066	1.5849	1.2823	1.8197	1.0471	1.2246	1.2246	1.406	0.9036	0.7516	1.0864	0.8872	1.19±0.29	0.0492
91	BB_0519	grpE protein (grpE)	1.3183	1.7539	0.6668	0.8872	1.3677	1.4859	0.7178	0.7727	2.421	1.0471	1.2706	0.5808	1.19±0.51	0.241

132	BB_0215	phosphate ABC transporter periplasmic phosphate-binding protein (pstS)	1.0666	1.3183	0.9036	1.1066	1.1803	1.3305	0.9908	1.1272	1.2942	1.6293	1.0965	1.3804	1.2±0.19	0.0047
116	BB_0028	predicted coding region BB0028	1.3305	1.3932	0.955	1.0568	1.5136	1.977	1.1272	1.4588	1.2246	0.8166	0.871	0.6546	1.2±0.35	0.0863
229	BB_0446	aspartyl-tRNA synthetase (aspS)	0.7516	0.7379	1.1169	1.0864	1.0186	1.2134	1.5417	2.0701	0.7311	1.2023	1.0765	1.9055	1.2±0.42	0.1313
53	BB_0067	peptidase putative	1.0864	1.3932	1.2134	1.5136	1.2589	1.3428	1.3804	1.4723	0.8395	0.9817	0.929	1.0864	1.21±0.21	0.0078
180	BB_0190	translation initiation factor 3 (infC)	1.3305	1.6596	1.2023	1.4859	1.1272	1.0375	1.0093	0.9204	1.3804	1.2023	1.2359	1.0765	1.22±0.2	0.0041
261	BB_0260	predicted coding region BB0260	0.7943	1.6596	0.6252	1.2359	1.2942	1.5276	0.9462	1.1066	2.4434	0.7047	1.8535	0.5012	1.22±0.55	0.2013
347	BB_0416	pheromone shutdown protein (traB)	1.1912	1.2823	1.4997	1.6293	0.955	1.0093	1.2023	1.2706	0.9908	1.0765	1.2474	1.3677	1.23±0.2	0.0028
353	BB_E16	predicted coding region BBE16	1.2359	1.8197	1.3305	1.9409	1.1803	1.5276	1.2823	1.6293	1.0864	0.2355	1.1803	0.2704	1.23±0.5	0.1641
39	BB_0730	glucose-6-phosphate isomerase (pgi)	1.2359	1.3062	1.9409	2.0324	1.1169	0.9727	1.7378	1.5136	0.8318	0.2992	1.2706	0.4742	1.23±0.51	0.1698
134	BB_0084	aminotransferase (nifS)	0.7943	1.5136	0.955	1.9588	1.1912	1.406	1.4997	1.7865	1.3804	0.2109	1.8197	0.2679	1.23±0.55	0.1892
103	BB_0479	ribosomal protein L4 (rplD)	2.1281	1.8535	1.7865	1.5417	0.9727	1.8365	0.8872	1.5849	0.4571	0.7047	0.4365	0.5916	1.23±0.59	0.2199
257	BB_A21	conserved hypothetical protein	1.2589	1.3677	1.2474	1.3552	1.0965	1.1695	1.0864	1.1588	1.1803	1.3804	1.1695	1.3677	1.24±0.1	0.0001
47	BB_K01	predicted coding region BBK01	1.028	1.2706	1.3428	1.5417	0.9036	1.1588	1.028	1.406	1.6144	0.6668	2.0137	0.8472	1.24±0.36	0.0535
296	BB_0567	chemotaxis histidine kinase (cheA-1)	1.5136	0.8166	2.1677	1.1588	1.3932	0.9204	2.0137	1.3062	0.7379	0.7244	1.0568	1.028	1.24±0.45	0.1103
350	BB_0500	ribosomal protein S13 (rpsM)	1.3428	1.1066	1.3677	1.1169	1.3428	1.2706	1.3677	1.2823	1.0666	1.3183	1.0864	1.3428	1.25±0.11	0.0001
158	BB_0612	ATP-dependent Clp protease subunit X (clpX)	1.3428	1.888	1.2589	1.7061	1.028	0.9462	0.912	0.8551	1.1376	1.5276	1.0093	1.3552	1.25±0.32	0.0247
143	BB_K45	immunogenic protein P37 putative	1.4454	1.0375	1.3677	0.9727	1.4997	1.4997	1.4191	1.406	1.6444	0.6194	1.5276	0.5861	1.25±0.34	0.0335
128	BB_0628	conserved hypothetical protein	0.9817	1.6904	1.0375	1.7539	1.2942	1.406	1.3428	1.4588	1.6596	0.3908	1.7219	0.3873	1.26±0.46	0.0859
239	BB_0296	heat shock protein (hslV)	1.3183	1.3804	1.3062	1.3305	1.3062	1.2706	1.2706	1.2359	0.9727	1.4723	0.955	1.4322	1.27±0.15	0.0001
282	BB_0042	phosphate transport system regulatory protein (phoU)	1.1912	0.9727	1.0186	0.863	1.5417	1.1912	1.3677	1.0765	0.9817	2.1878	0.8551	1.9588	1.27±0.41	0.0539
339	BB_F24	conserved hypothetical protein	1.2134	1.2246	1.3428	1.3552	1.1588	0.8872	1.2823	0.9817	1.2589	1.5276	1.406	1.6904	1.28±0.21	0.001
292	BB_0341	glu-tRNA amidotransferase subunit B (gatB)	1.1482	1.1376	1.1695	1.1695	1.4859	1.3183	1.5136	1.3428	1.2942	1.2942	1.3183	1.3183	1.29±0.12	0.0001
428	BB_J23	predicted coding region BBJ23	1.7701	1.3552	1.1272	0.863	1.5417	1.7061	0.9817	1.0965	1.2942	1.803	0.8241	1.1482	1.29±0.33	0.0136
107	BB_0220	alanyl-tRNA synthetase (alaS)	1.3183	1.4454	1.8197	1.9953	1.0375	1.0765	1.4723	1.4997	0.929	0.6138	1.3183	0.9036	1.29±0.38	0.0296
60	BB_A69	predicted coding region BBA69	1.0186	1.0375	1.406	1.3804	1.0965	1.0765	1.5276	1.4322	2.1281	0.2535	2.8054	0.2965	1.29±0.67	0.1811
247	BB_0487	ribosomal protein S17 (rpsQ)	1.4322	1.2706	1.6596	1.4454	1.3804	1.1695	1.5704	1.3305	1.4588	0.5861	1.6293	0.6668	1.3±0.33	0.0119
388	BB_0481	ribosomal protein L2 (rplB)	1.3305	1.0093	1.1695	0.8872	1.5276	1.406	1.3428	1.2359	1.4997	1.5704	1.3183	1.3804	1.31±0.2	0.0003
402	BB_0651	conserved hypothetical protein	1.7061	2.0893	1.4322	1.7219	1.4322	1.5417	1.1912	1.2706	1.7701	0.0449	1.4588	0.0398	1.31±0.61	0.1225
89	BB_0478	ribosomal protein L3 (rplC)	1.3804	1.7219	1.0471	1.2823	1.6444	1.7219	1.2246	1.2823	2.0512	0.5495	1.556	0.4246	1.32±0.46	0.0384
185	BB_0158	antigen S2 putative	1.2942	1.2474	1.4588	1.406	1.1695	1.2134	1.3183	1.3677	1.4322	1.1803	1.5996	1.3183	1.33±0.12	0.0001
212	BB_0494	ribosomal protein L18 (rplR)	1.6904	1.3804	2.1281	1.7219	1	1.1803	1.2589	1.4723	0.9376	0.8551	1.1695	1.1272	1.33±0.36	0.0113
216	BB_0483	ribosomal protein L22 (rplV)	1.803	1.4859	1.5996	1.3183	2.0701	1.4191	1.8365	1.2706	1.1066	0.5702	0.9817	0.5012	1.33±0.46	0.0374
366	BB_0188	ribosomal protein L20 (rplT)	1.5417	1.1169	1.4859	1.0864	1.4322	1.3305	1.3804	1.2823	1.1588	1.5704	1.1272	1.5136	1.34±0.17	0.0001
52	BB_0593	long-chain-fatty-acid CoA ligase	1.2474	1.5417	1.5704	1.9409	1.1169	1.028	1.406	1.2942	1.1376	1.0568	1.4588	1.3552	1.35±0.25	0.0008
15	BB_0540	translation elongation factor G (fus-1)	1.5704	2.2284	1.4588	2.1478	1.5276	1.406	1.4191	1.2942	0.912	0.7447	0.863	0.6792	1.35±0.48	0.0322
123	BB_0490	ribosomal protein L5 (rplE)	1.1588	1.6444	1.4859	2.0512	1.0568	1.1803	1.3552	1.4723	1.6749	0.4831	2.0893	0.5808	1.35±0.48	0.0328
157	BB_0495	ribosomal protein S5 (rpsE)	1.2023	1.3552	1.1695	1.3183	1.3428	1.3804	1.2942	1.3305	1.4191	1.6144	1.3552	1.5417	1.36±0.12	0.0001
297	BB_0338	ribosomal protein S9 (rpsI)	1.5136	1.4723	1.1169	1.0666	1.6144	1.6596	1.1803	1.2134	1.6596	1.4859	1.2246	1.0666	1.36±0.22	0.0003
167	BB_0501	ribosomal protein S11 (rpsK)	1.7219	1.4723	1.6444	1.4588	1.3552	1.3677	1.3305	1.3305	0.8241	1.5136	0.8091	1.4859	1.36±0.27	0.001
19	BB_0056	phosphoglycerate kinase (pgk)	1.2359	2.0512	1.0093	1.7061	2.0324	1.8535	1.6904	1.5276	1.0375	0.7379	0.863	0.597	1.36±0.49	0.0325
278	BB_E31	antigen P35 putative	2.421	2.2491	1.5849	1.5276	1.5704	1.7539	1.0965	1.2474	0.5702	1.1376	0.4055	0.787	1.36±0.59	0.0666
83	BB_0727	pyrophosphate--fructose 6-phosphate 1-phosphotransferase (pfk)	1.2706	1.4322	1.3552	1.5276	1.3062	1.3552	1.3932	1.4454	1.406	1.2823	1.4997	1.3932	1.39±0.08	0.0001
139	BB_0492	ribosomal protein S8 (rpsH)	1.3932	1.6749	1.1272	1.3428	1.5276	1.4859	1.2246	1.2023	1.3552	1.803	1.0765	1.4588	1.39±0.21	0.0001
211	BB_0168	dnaK suppressor putative	1.2246	1.5849	1.4859	1.8707	1.1169	1.0864	1.2359	1.2823	1.9055	0.7727	2.208	0.8872	1.39±0.42	0.0102
98	BB_0093	V-type ATPase subunit B (atpB)	1.7701	1.7539	1.7701	1.7378	1.3552	1.4997	1.3552	1.4997	0.863	1.1912	0.8318	1.1803	1.4±0.32	0.0017
233	BB_J36	predicted coding region BBJ36	1.3552	1.2823	1.3428	1.2942	1.4588	1.3804	1.4588	1.406	1.8197	1.1272	1.8707	1.1169	1.41±0.22	0.0001
171	BB_0588	pfs protein (pfs-2)	1.0765	1.406	0.7047	0.8872	1.556	2.1086	1.0186	1.4454	2.5119	1.556	1.7378	0.9638	1.41±0.51	0.0203
33	BB_0123	ribosomal protein S2 (rpsB)	1.5704	1.6293	1.2134	1.2823	1.6444	1.5996	1.2823	1.2589	2.2909	0.8395	1.7865	0.6368	1.42±0.42	0.0067
252	BB_0699	ribosomal protein L19 (rplS)	1.4997	1.5704	1.2706	1.3305	1.7378	1.4454	1.4588	1.2246	1.5417	1.5136	1.2942	1.2246	1.43±0.15	0.0001
118	BB_0094	V-type ATPase subunit A (atpA)	1.9055	2.0324	1.6444	1.7865	1.8197	1.4588	1.6144	1.2942	1.3804	0.5297	1.2589	0.4786	1.43±0.48	0.0116
277	BB_0503	ribosomal protein L17 (rplQ)	1.5704	1.5417	0.871	0.8551	2.0512	1.9953	1.1376	1.1066	2.355	1.5417	1.3062	0.8551	1.43±0.48	0.0126
147	BB_H06	predicted coding region BBH06	1.977	1.6596	0.9638	0.871	2.6546	2.1281	1.3428	1.0765	2.3988	0.7244	1.1482	0.3373	1.44±0.69	0.0579

411	BB_0818	conserved hypothetical protein	1.3305	0.8241	2.421	1.4997	1.0186	0.9727	1.8535	1.7701	0.7943	1.2359	1.4454	2.2491	1.45±0.51	0.0139
7	BB_0147	flagellar filament 41 kDa core protein (flaB)	0.7447	1.8707	0.3221	0.8241	3.2509	2.6546	1.4454	1.1695	2.4889	1.1803	1.0666	0.5445	1.46±0.88	0.1074
73	BB_0749	predicted coding region BB0749	0.8017	1.3552	1.1066	1.7378	1.4588	1.5136	1.803	1.888	1.406	1.2823	1.7701	1.5849	1.48±0.3	0.0003
250	BB_K40	conserved hypothetical protein	1.3677	1.4859	1.2823	1.3677	1.6293	1.6596	1.4997	1.5276	1.8707	1.4997	1.7219	1.3804	1.52±0.16	0.0001
119	BB_0713	conserved hypothetical protein	1.6144	1.9409	1.0186	1.2023	1.8197	2.0701	1.1376	1.2942	2.3335	1.4322	1.4454	0.879	1.52±0.43	0.0022
62	BB_0122	translation elongation factor TS (tsf)	1.5849	2.0324	1.7539	2.3121	1.4997	1.4454	1.6749	1.6293	0.6546	1.3677	0.7178	1.5136	1.52±0.45	0.0029
191	BB_0742	ABC transporter ATP-binding protein	1.5276	1.6904	1.5276	1.6749	1.7219	1.3804	1.7061	1.3804	1.1912	1.803	1.1588	1.7701	1.54±0.21	0.0001
304	BB_0541	predicted coding region BB0541	1.2589	1.6749	0.955	1.2706	1.6444	1.2823	1.2942	0.9817	2.421	2.1878	1.803	1.803	1.55±0.44	0.0016
371	BB_0232	hbbU protein	1.3932	1.5849	2.5351	2.8054	1.3552	1.3552	2.421	2.466	0.8872	0.0391	1.6293	0.0745	1.55±0.87	0.0626
65	BB_0833	isoleucyl-tRNA synthetase (ileS)	1.5276	1.5996	2.1086	2.1878	1.2134	1.0568	1.6904	1.4588	1.2474	1.1695	1.7219	1.7061	1.56±0.34	0.0002
206	BB_0689	predicted coding region BB0689	2.1281	1.9588	2.0512	1.8197	1.4454	1.4859	1.406	1.4191	1.3932	1.2823	1.3062	1.2023	1.57±0.31	0.0001
135	BB_0109	acetyl-CoA C-acetyltransferase (fadA)	1.2706	1.5276	1.3062	1.5704	1.803	1.3677	1.8365	1.406	2.0324	1.406	2.0701	1.4322	1.59±0.27	0.0001
196	BB_0105	methionine aminopeptidase (map)	1.0864	2.0137	1.1912	2.2699	1.7539	1.4723	1.9588	1.6904	2.1086	0.6138	2.355	0.6792	1.6±0.57	0.005
77	BB_0484	ribosomal protein S3 (rpsC)	1.9588	2.0893	1.3932	1.4859	2.1677	1.803	1.5276	1.2823	1.4859	1.888	1.028	1.3428	1.62±0.34	0.0001
199	BB_0691	translation elongation factor G (fus-2)	0.912	1.6749	0.9204	1.5704	2.2491	1.9953	2.2284	1.977	1.7061	1.4723	1.7061	1.4859	1.66±0.42	0.0003
310	BB_0802	ribosome-binding factor A (rbfA)	2.466	2.6062	1.5417	1.6904	1.3305	2.1086	0.7798	1.3305	1.9588	1.7378	1.2474	1.0864	1.66±0.53	0.0017
184	BB_0355	transcription factor putative	1.0375	1.7378	0.6668	1.1588	1.7061	2.1878	1.1588	1.4723	2.3768	2.9376	1.6144	1.9588	1.67±0.61	0.0039
50	BB_0690	neutrophil activating protein (napA)	1.4997	1.3183	0.9638	0.8472	2.7542	3.3113	1.7865	2.1878	2.3121	1.0186	1.5136	0.673	1.68±0.78	0.0146
140	BB_0251	leucyl-tRNA synthetase (leuS)	2.2699	1.9953	2.1086	1.8197	2.0893	1.4588	1.9588	1.3677	1.4859	1.4191	1.4322	1.3932	1.73±0.32	0.0001
159	BB_0386	ribosomal protein S7 (rpsG)	2.1677	2.1677	1.4859	1.4859	2.2284	1.8707	1.5276	1.2706	1.8707	2.0893	1.2706	1.4322	1.74±0.35	0.0001
193	BB_0513	phenylalanyl-tRNA synthetase alpha subunit (pheS)	1.2823	1.1272	2.729	2.3988	1.0965	1.1482	2.4889	2.5823	0.7447	1.0186	1.8365	2.421	1.74±0.71	0.0053
85	BB_J34	predicted coding region BBJ34	2.1281	3.1915	1.3183	1.9409	1.3183	2.1478	0.8395	1.3062	2.9648	1.1912	1.8197	0.7447	1.74±0.74	0.0069
84	BB_0132	transcription elongation factor (greA)	3.1046	2.466	2.2284	1.8197	1.3428	1.8707	0.9908	1.3552	2.3335	1.1066	1.7865	0.9036	1.78±0.64	0.0021
28	BB_0435	DNA gyrase subunit A (gyrA)	1.9588	2.5586	1.5704	2.1478	1.9055	2.3335	1.556	1.9409	1.4322	1.556	1.1912	1.2823	1.79±0.41	0.0001
76	BB_0436	DNA gyrase subunit B (gyrB)	1.2246	1.5417	2.0893	2.5586	1.5704	1.4588	2.5586	2.4434	1.3305	0.9376	2.2491	1.5849	1.8±0.53	0.0004
8	BB_0560	heat shock protein 90 (htpG)	2.0893	2.466	1.4859	2.0324	2.1677	2.6792	1.7061	2.0701	2.0893	0.6427	1.6749	0.5248	1.8±0.63	0.0014
90	BB_0154	preprotein translocase subunit (secA)	1.3062	1.1169	2.9923	2.5351	0.9376	1.1272	2.1086	2.5351	1.1588	1.2474	2.5823	2.7797	1.87±0.75	0.0027
141	BB_0438	DNA polymerase III subunit beta (dnaN)	1.0864	1.2474	2.8576	3.1623	1.2942	1.1803	3.2211	2.9923	0.673	0.871	1.6904	2.2909	1.88±0.92	0.0089
4	BB_0057	glyceraldehyde 3-phosphate dehydrogenase (gap)	1.4454	2.466	0.7798	1.406	4.1687	3.5318	2.3335	2.0512	2.1677	0.7178	1.2359	0.3981	1.89±1.09	0.02
168	BB_0579	DNA polymerase III subunit alpha (dnaE)	1.803	1.5996	2.2284	2.0137	1.977	1.5704	2.355	1.9055	1.7701	1.6144	2.1086	2.0137	1.91±0.24	0.0001
72	BB_0444	nucleotide sugar epimerase	1.5996	2.3768	1.6596	2.5351	2.1878	1.7378	2.3335	1.8365	2.208	1.0765	2.3335	1.028	1.91±0.48	0.0001
42	BB_0295	heat shock protein (hslU)	2.2909	2.729	1.977	2.4889	1.8197	2.3988	1.6444	2.2284	1.8707	0.9638	1.7865	0.8872	1.92±0.54	0.0001
336	BB_0256	ribosomal protein S21 (rpsU)	2.355	1.9231	1.4859	1.1912	2.2491	2.355	1.3932	1.4723	2.729	2.6303	1.6904	1.6293	1.93±0.5	0.0001
58	BB_0392	ribosomal protein L1 (rplA)	1.9409	2.6792	1.3932	1.9231	2.0893	2.3768	1.4997	1.7061	2.5119	1.9231	1.803	1.3932	1.94±0.4	0.0001
117	BB_0683	3-hydroxy-3-methylglutaryl-CoA synthase	1.9231	1.977	2.0324	2.0701	2.1878	1.888	2.2699	1.9953	1.6749	1.7539	1.7539	1.8707	1.95±0.17	0.0001
59	BB_0712	RNA polymerase sigma-70 factor (rpoD)	1.7219	1.3428	2.2699	1.7219	2.2699	2.3768	2.8576	2.884	1.1066	1.6596	1.4191	1.9953	1.97±0.55	0.0001
30	BB_0558	phosphoenolpyruvate-protein phosphatase (ptsI)	1.5704	2.1281	2.0137	2.7797	2.3335	1.8365	2.9923	2.3768	1.2589	1.1376	1.6904	1.4859	1.97±0.56	0.0001
352	BB_0454	lipopolysaccharide biosynthesis-related protein	1.0965	1.5704	2.0512	2.9376	0.8318	1.3932	1.5704	2.6303	1.7378	1.5704	3.2509	2.9648	1.97±0.76	0.0014
32	BB_A03	outer membrane protein	1.3305	2.0893	1.7061	2.729	1.406	1.8365	1.7539	2.3768	3.1623	0.7516	3.9084	0.9204	2±0.88	0.0032
224	BB_0652	protein-export membrane protein (secD)	1.2589	0.8318	3.2211	2.0324	1.2474	1.4723	3.1623	3.7325	1.2589	0.673	3.2509	1.803	2±1.02	0.008
56	BB_0230	transcription termination factor Rho (rho)	2.1086	2.2284	2.2491	2.3768	1.7061	1.8365	1.8197	1.9588	1.8197	2.1086	1.9409	2.2284	2.03±0.2	0.0001
312	BB_0578	methyl-accepting chemotaxis protein (mcp-1)	1.9953	2.1281	2.0512	2.1677	1.5996	1.8535	1.6444	1.9055	1.7219	2.8054	1.803	2.8314	2.04±0.39	0.0001
181	BB_0339	ribosomal protein L13 (rplM)	2.4889	3.4041	1.3552	2.0137	2.9648	2.5119	1.7061	1.3428	1.6749	2.9648	0.9462	1.5417	2.08±0.74	0.0005
40	BB_A16	outer surface protein B (ospB)	1.0568	1.2134	2.3335	2.6303	1.3183	1.556	2.8576	3.3729	1.5704	1.4454	3.3729	3.02	2.15±0.84	0.0009
188	BB_0115	ribosomal protein S6 (rpsF)	1.5417	1.7378	2.3335	2.704	1.8365	1.7378	2.7797	2.6546	1.803	2.3121	2.8054	3.5318	2.31±0.57	0.0001
13	BB_0805	polyribonucleotide nucleotidyltransferase (pnpA)	3.4041	3.3729	1.6444	1.6596	3.8726	3.6308	1.8365	1.7701	2.729	1.888	1.4322	0.9462	2.35±0.95	0.0006
202	BB_0114	single-stranded DNA-binding protein (ssb)	2.884	2.4434	1.5276	1.4588	2.5119	2.8576	1.4322	1.6293	3.1333	4.3652	1.7061	2.5351	2.37±0.84	0.0002
214	BB_0113	ribosomal protein S18 (rpsR)	3.1333	4.0179	1.6749	2.1478	3.3729	3.2509	1.803	1.7219	4.0926	2.2284	2.208	1.028	2.56±0.95	0.0002
93	BB_0390	ribosomal protein L7/L12 (rplL)	3.0479	3.4041	1.2589	1.4322	4.2462	4.0179	1.7701	1.7061	4.1305	3.4041	1.7378	1.3804	2.63±1.13	0.0006
207	BB_0776	predicted coding region BB0776	3.767	2.3335	3.8019	2.3988	3.4356	4.1305	3.8019	4.2462	0.7798	1.5276	0.7727	1.6293	2.72±1.25	0.0008
96	BB_0615	ribosomal protein S4 (rpsD)	3.1046	2.8576	3.02	2.7542	3.3729	2.6546	3.2211	2.5823	2.884	1.8197	2.8054	1.7539	2.74±0.48	0.0001
152	BB_0696	conserved hypothetical protein	3.3729	5.3456	1.5849	2.5823	5.2481	6.0256	2.6792	3.1333	3.1046	2.0137	1.6904	1.1912	3.16±1.52	0.0006

5	BB_0388	DNA-directed RNA polymerase (rpoC)	1.7539	1.4859	5.2	4.7424	1.3932	1.4454	4.4463	4.5709	1.7865	1.7865	5.2481	5.1523	3.25±1.66	0.0009
78	BB_0504	conserved hypothetical protein	3.6644	4.4875	3.2509	4.0179	2.6062	3.3729	2.3335	3.02	4.2855	3.6983	3.8371	3.3113	3.49±0.62	0.0001
108	BB_0393	ribosomal protein L11 (rplK)	3.7325	5.6494	1.8707	3.1915	6.0256	5.1523	3.4995	2.8314	4.7863	1.5849	2.8054	1.0093	3.51±1.55	0.0002
3	BB_0389	DNA-directed RNA polymerase (rpoB)	3.2211	2.355	6.1944	4.9204	3.0761	3.02	6.0256	6.368	3.1333	2.2699	6.1376	4.4055	4.26±1.53	0.0001
63	BB_A68	predicted coding region BBA68	3.3419	4.0179	4.0551	4.9659	3.8019	4.0179	4.6132	4.9659	5.0119	2.8576	6.0813	3.4995	4.27±0.85	0.0001
34	BB_0502	DNA-directed RNA polymerase (rpoA)	6.4269	8.7096	2.5119	3.9084	7.5162	7.1779	3.3113	2.8314	5.4954	5.0119	2.3121	2.1281	4.78±2.18	0.0001
27	BB_B19	outer surface protein C (ospC)	21.0863	18.0302	1.1376	1.0666	19.4089	16.9044	1.0186	0.879	14.9969	11.0662	0.6855	0.5754	8.9±8.33	0.0093
57	BB_0443	spoIIIJ-associtated protein (jag)	13.4277	13.3045	7.9433	7.9433	14.7231	13.0617	8.091	7.7268	17.378	9.4624	10.5682	5.8076	10.79±3.38	0.0001
20	BB_I39	predicted coding region BBI39	10.5682	10.4713	14.0605	14.1906	9.2045	8.9536	13.4277	12.3595	11.9124	6.8549	16.1436	9.8175	11.5±2.55	0.0001
99	BB_A24	decorin binding protein A (dbpA)	27.0396	28.5759	1.7701	2.0701	23.3346	22.9087	1.5136	1.4588	18.7068	12.1339	1.0186	0.7244	11.77±11.05	0.008

The iTRAQ labels used were: 113 and 117 for wild-type *B.burgdorferi* , 114 and 118 for *hrpA2* (GCB1164), 115 and 119 for *hrpA3* (GCB1165), 116 and 121 for *hrpA4* (GCB1166). The columns between D and O represents the iTRAQ quantification ratios of the *hrpA* mutant strains over wild-type. Results are ranked according to increasing mean values. P values were calculated based upon the 12 input results using a two-sided, one sample t-test with comparison against a theoretical value of 1.0. Colored rows indicate proteins with P values greater than 0.05, which were excluded from Tables 4 and 5. See Materials and Methods for further details.
