# Supplementary material for: HrpA, a DEAH-Box RNA Helicase, Is Involved in Global Gene Regulation in the Lyme Disease Spirochete
Source: PLoS One. 2011 Jul 26;6(7):e22168. doi: 10.1371/journal.pone.0022168 (PMC3144200; doi:10.1371/journal.pone.0022168)
Supplement: Table S3 — Downregulated B. burgdorferi proteins in hrpA mutant clones compared to wild-type based upon iTRAQ analysis. (PDF) [file pone.0022168.s005.pdf]

Table S3. Downregulated *B. burgdorferi* proteins in *hrpA* mutant clones compared to wild-type based upon iTRAQ analysis

ORF	Protein Name	Mean ± SD	P value	Decrease
BB_0241	GLPK	0.07±0.03	0.0001	14.28
BB_0243	GLPA	0.11±0.02	0.0001	9.09
BB_0603	Membrane-assoc. prot p66	0.19±0.07	0.0001	5.2
BB_A74	Outer membrane porin	0.25±0.18	0.0001	4
BB_0330	OppA-3	0.29±0.1	0.0001	3.44
BB_B29	PTS malt and gluc ABC comp	0.29±0.1	0.0001	3.44
BB_0365	Antigen IPLA7	0.3±0.14	0.0001	3.33
BB_B07	Outer surface prot., putative	0.32±0.11	0.0001	3.12
BB_0328	OppA-1	0.34±0.16	0.0001	2.94
BB_0337	Enolase	0.35±0.06	0.0001	2.85
BB_0238	Predicted coding region	0.37±0.11	0.0001	2.7
BB_J09	OspD	0.38±0.18	0.0001	2.63
BB_0476	Translation elong factor TU	0.4±0.13	0.0001	2.5
BB_0744	Antigen p83/100	0.41±0.17	0.0001	2.43
BB_B06	PTS cellob. IIB component	0.43±0.04	0.0001	2.32
BB_0622	Acetate kinase	0.44±0.16	0.0001	2.27
BB_0210	Surface-located memb prot1	0.45±0.17	0.0001	2.22
BB_I29	Predicted protein	0.46±0.18	0.0001	2.17
BB_B05	PTS cellob IIA component	0.47±0.31	0.0002	2.12
BB_0408	PTS fruct IIABC component	0.48±0.16	0.0001	2.08
BB_0020	PF6P1P β sub	0.49±0.17	0.0001	2.04
BB_0204	Lambda CII stability-gov prot (HflC)	0.49±0.17	0.0001	2.04
BB_0329	OppA-2	0.49±0.17	0.0001	2.04
BB_0385	Basic membrane prot D	0.5±0.17	0.0001	2
BB_0383	Basic membrane prot A	0.51±0.19	0.0001	1.96
BB_0426	Predicted protein	0.51±0.24	0.0001	1.96
BB_0517	Chaperone protein dnaJ	0.51±0.12	0.0001	1.96
BB_0649	60 kDa chaperonin (groEL)	0.52±0.29	0.0002	1.92
BB_K47	Predicted protein	0.52±0.22	0.0001	1.92
BB_0323	Predicted protein	0.54±0.05	0.0001	1.85
BB_0608	Aminoacyl-his dipeptidase	0.54±0.14	0.0001	1.85
BB_0659	Lysyl-tRNA synthetase	0.56±0.17	0.0001	1.78
BB_0375	Pfs prot	0.57±0.17	0.0001	1.75
BB_0462	Predicted protein	0.57±0.12	0.0001	1.75
BB_0024	Predicted protein	0.58±0.18	0.0001	1.72
BB_0668	Flag filament outer layer prot	0.58±0.25	0.0002	1.72
BB_0536	Zinc protease putative	0.6±0.11	0.0001	1.66
BB_0741	10 kDa chaperonin (groES)	0.6±0.2	0.0001	1.66
BB_0334	OppD	0.61±0.13	0.0001	1.63
BB_B16	OppAIV	0.61±0.29	0.0009	1.63
BB_0068	Predicted protein	0.62±0.11	0.0001	1.61
BB_O33	Predicted protein	0.62±0.12	0.0001	1.61
BB_0364	Predicted protein	0.63±0.08	0.0001	1.58
BB_B22	Predicted protein	0.63±0.12	0.0001	1.58
BB_0137	Longchain-fatty-acid CoA lig	0.64±0.15	0.0001	1.56
BB_0153	Superoxide dismutase	0.64±0.11	0.0001	1.56
BB_0195	Cell div. control prot 27, putative	0.64±0.04	0.0001	1.56
BB_0488	Ribosomal prot L14	0.64±0.1	0.0001	1.56

BB_B28	Predicted protein	0.64±0.21	0.0001	1.56
BB_0653	Protein-export memb prot. SecF	0.65±0.05	0.0001	1.53
BB_K13	Predicted protein	0.65±0.33	0.0041	1.53
BB_0087	L-lactate dehydrogenase	0.66±0.21	0.0002	1.51
BB_0203	Lambda CII stability-gov prot (HflK)	0.67±0.14	0.0001	1.49
BB_0069	Aminopeptidase II	0.68±0.17	0.0001	1.47
BB_0151	N-acetylglucos-6-P deacetylase	0.68±0.25	0.0016	1.47
BB_0226	Seryl-tRNA synthetase	0.68±0.19	0.0001	1.47
BB_0227	Predicted protein	0.68±0.16	0.0001	1.47
BB_0366	Aminopeptidase I	0.68±0.34	0.0095	1.47
BB_0463	Nucleoside-diphosph kinase	0.68±0.17	0.0001	1.47
BB_0537	Predicted protein	0.68±0.3	0.0044	1.47
BB_0761	Prot chemotax resp.reg, putative	0.68±0.16	0.0001	1.47
BB_P38	ErpA	0.69±0.13	0.0001	1.47
BB_0445	Fruc-bisphosph aldolase	0.69±0.32	0.0088	1.44
BB_0642	Sperm/putresc ABC transport	0.7±0.31	0.0086	1.44
BB_0335	OppF	0.71±0.17	0.0002	1.42
BB_0300	Cell division prot	0.71±0.23	0.0016	1.4
BB_0551	CheY-1	0.72±0.16	0.0001	1.4
BB_0606	Predicted protein	0.73±0.14	0.0001	1.38
BB_0034	Predicted protein	0.73±0.19	0.0006	1.36
BB_0449	Predicted protein	0.73±0.25	0.004	1.36
BB_0553	Predicted protein	0.73±0.23	0.002	1.36
BB_B18	GMP synthase	0.73±0.2	0.0001	1.36
BB_L34	Predicted protein	0.73±0.25	0.0038	1.36
BB_Q34	BdrW	0.74±0.06	0.0001	1.36
BB_0376	MetK	0.75±0.14	0.0001	1.35
BB_0103	Predicted protein	0.76±0.13	0.0001	1.33
BB_0047	Predicted protein	0.76±0.36	0.0476	1.31
BB_0405	Predicted protein	0.76±0.34	0.0423	1.31
BB_0546	Predicted protein	0.76±0.14	0.0001	1.31
BB_0695	Ribosomal prot S16	0.76±0.28	0.0149	1.31
BB_0752	Predicted protein	0.77±0.13	0.0001	1.31
BB_0840	Lipoprotein, putative	0.78±0.13	0.0001	1.29
BB_0169	Translation initiat factor 1	0.79±0.27	0.0257	1.28
BB_0325	Predicted protein	0.78±0.17	0.0014	1.28
BB_B17	IMP dehydrogenase	0.78±0.32	0.0455	1.28
BB_0705	Ribonuclease III	0.82±0.05	0.0001	1.26
BB_0194	Predicted protein	0.82±0.22	0.0211	1.21
BB_0575	CTP synthase	0.82±0.05	0.0001	1.21
BB_0599	Cysteinyl-tRNA synthetase	0.83±0.06	0.0001	1.21
BB_F20	Predicted protein	0.83±0.13	0.0012	1.2

Mean values and standard deviations were determined using 12 input results obtained from iTRAQ quantification values. P values were calculated based upon the 12 input results using a two-sided, one sample *t* test with comparison against a theoretical value of 1.0.
Colored cells contain proteins whose expression is reported to be regulated only by HrpA.. The complete list of proteins identified in the iTRAQ experiment is given in **Table S2**.
