# Supplementary material for: HrpA, a DEAH-Box RNA Helicase, Is Involved in Global Gene Regulation in the Lyme Disease Spirochete
Source: PLoS One. 2011 Jul 26;6(7):e22168. doi: 10.1371/journal.pone.0022168 (PMC3144200; doi:10.1371/journal.pone.0022168)
Supplement: Table S4 — Upregulated B. burgdorferi proteins in hrpA mutant clones compared to wild-type, based upon iTRAQ analysis. (PDF) [file pone.0022168.s006.pdf]

Table S4. Upregulated *B. burgdorferi* proteins in *hrpA* mutant clones compared to wild-type, based upon iTRAQ analysis

ORF	Protein Name	Mean ± StdDev	P value	Increase
BB_A24	Decorin binding prot A	11.77±11.05	0.008	11.77
BB_I39	Predicted protein	11.5±2.55	0.0001	11.5
BB_0443	Jag	10.79±3.38	0.0001	10.79
BB_B19	OspC	8.9±8.33	0.0093	8.9
BB_0502	RpoA	4.78±2.18	0.0001	4.78
BB_A68	Predicted protein	4.27±0.85	0.0001	4.27
BB_0389	RpoB	4.26±1.53	0.0001	4.26
BB_0393	Ribosomal prot L11	3.51±1.55	0.0002	3.51
BB_0504	Predicted protein	3.49±0.62	0.0001	3.49
BB_0388	RpoC	3.25±1.66	0.0009	3.25
BB_0696	Predicted protein	3.16±1.52	0.0006	3.16
BB_0615	Ribosomal prot S4	2.74±0.48	0.0001	2.74
BB_0776	Predicted protein	2.72±1.25	0.0008	2.72
BB_0390	Ribosomal prot L7/L12	2.63±1.13	0.0006	2.63
BB_0113	Ribosomal prot S18	2.56±0.95	0.0002	2.56
BB_0114	SSB	2.37±0.84	0.0002	2.37
BB_0805	Polyribonucleotide transferase	2.35±0.95	0.0006	2.35
BB_0115	Ribosomal prot S6	2.31±0.57	0.0001	2.31
BB_A16	OspB	2.15±0.84	0.0009	2.15
BB_0339	Ribosomal prot L13	2.08±0.74	0.0005	2.08
BB_0578	Mcp-1	2.04±0.39	0.0001	2.04
BB_0230	Transcription term fact Rho	2.03±0.2	0.0001	2.03
BB_0652	Protein-export membrane prot secD	2±1.02	0.008	2
BB_A03	Outer membrane prot	2±0.88	0.0032	2
BB_0454	Lipopolysacch biosynth prot	1.97±0.76	0.0014	1.97
BB_0558	Phosphoenolpyruvate phosph	1.97±0.56	0.0001	1.97
BB_0712	RpoD	1.97±0.55	0.0001	1.97
BB_0683	3-OH-3-methylglut-CoA synth	1.95±0.17	0.0001	1.95
BB_0392	Ribosomal prot L1	1.94±0.4	0.0001	1.94
BB_0256	Ribosomal prot S21	1.93±0.5	0.0001	1.93
BB_0295	Unfoldase HslU	1.92±0.54	0.0001	1.92
BB_0444	Nucleotide sugar epimerase	1.91±0.48	0.0001	1.91
BB_0579	DNA pol III subunit alpha	1.91±0.24	0.0001	1.91
BB_0057	G3P dehydrogenase	1.89±1.09	0.02	1.89
BB_0438	DNA pol III subunit beta	1.88±0.92	0.0089	1.88
BB_0154	Preprotein translocase subunit secA	1.87±0.75	0.0027	1.87
BB_0436	DNA gyrase subunit B	1.8±0.53	0.0004	1.8
BB_0560	Chaperone protein htpG	1.8±0.63	0.0014	1.8
BB_0435	DNA gyrase subunit A	1.79±0.41	0.0001	1.79
BB_0132	Transcription elong factor	1.78±0.64	0.0021	1.78
BB_0386	Ribosomal prot S7	1.74±0.35	0.0001	1.74
BB_0513	Phe-tRNA synth alpha subunit	1.74±0.71	0.0053	1.74
BB_J34	Predicted protein	1.74±0.74	0.0069	1.74
BB_0251	Leucyl-tRNA synthetase	1.73±0.32	0.0001	1.73
BB_0690	Neutrophil activating prot NapA	1.68±0.78	0.0146	1.68
BB_0355	Transcription factor putative	1.67±0.61	0.0039	1.67
BB_0691	Translation elong factor G	1.66±0.42	0.0003	1.66
BB_0802	Ribosome-binding factor A	1.66±0.53	0.0017	1.66
BB_0484	Ribosomal prot S3	1.62±0.34	0.0001	1.62
BB_0105	Methionine aminopeptidase	1.6±0.57	0.005	1.6
BB_0109	FadA	1.59±0.27	0.0001	1.59
BB_0689	Predicted protein	1.57±0.31	0.0001	1.57

BB_0833	Isoleucyl-tRNA synthetase	1.56±0.34	0.0002	1.56
BB_0541	Predicted protein	1.55±0.44	0.0016	1.55
BB_0742	ABC transport ATP-bind prot	1.54±0.21	0.0001	1.54
BB_0122	Translation elong factor TS	1.52±0.45	0.0029	1.52
BB_0713	Predicted protein	1.52±0.43	0.0022	1.52
BB_K40	Predicted protein	1.52±0.16	0.0001	1.52
BB_0749	Predicted protein	1.48±0.3	0.0003	1.48
BB_0818	Predicted protein	1.45±0.51	0.0139	1.45
BB_0094	V-type ATPase subunit A	1.43±0.48	0.0116	1.43
BB_0503	Ribosomal prot L17	1.43±0.48	0.0126	1.43
BB_0699	Ribosomal prot L19	1.43±0.15	0.0001	1.43
BB_0123	Ribosomal prot S2	1.42±0.42	0.0067	1.42
BB_0588	Pfs-2	1.41±0.51	0.0203	1.41
BB_J36	Predicted protein	1.41±0.22	0.0001	1.41
BB_0093	V-type ATPase subunit B	1.4±0.32	0.0017	1.4
BB_0168	DnaK suppressor, putative	1.39±0.42	0.0102	1.39
BB_0492	Ribosomal prot S8	1.39±0.21	0.0001	1.39
BB_0727	PFP phosphotransferase	1.39±0.08	0.0001	1.39
BB_0056	Phosphoglycerate kinase	1.36±0.49	0.0325	1.36
BB_0338	Ribosomal prot S9	1.36±0.22	0.0003	1.36
BB_0495	Ribosomal prot S5	1.36±0.12	0.0001	1.36
BB_0501	Ribosomal protS11	1.36±0.27	0.001	1.36
BB_0490	Ribosomal prot L5	1.35±0.48	0.0328	1.35
BB_0540	Translation elong factor G	1.35±0.48	0.0322	1.35
BB_0593	Long-chain-fatty-acid CoA ligase	1.35±0.25	0.0008	1.35
BB_0188	Ribosomal prot L20	1.34±0.17	0.0001	1.34
BB_0158	Antigen S2 putative	1.33±0.12	0.0001	1.33
BB_0483	Ribosomal prot L22	1.33±0.46	0.0374	1.33
BB_0494	Ribosomal prot L18	1.33±0.36	0.0113	1.33
BB_0478	Ribosomal prot L3	1.32±0.46	0.0384	1.32
BB_0481	Ribosomal prot L2	1.31±0.2	0.0003	1.31
BB_0487	Ribosomal prot S17	1.3±0.33	0.0119	1.3
BB_0220	AlaS	1.29±0.38	0.0296	1.29
BB_0341	Glu-tRNA amidotransf sub. B	1.29±0.12	0.0001	1.29
BB_J23	Predicted protein	1.29±0.33	0.0136	1.29
BB_F24	Predicted protein	1.28±0.21	0.001	1.28
BB_0296	ATP-dependent protease subunit HslV	1.27±0.15	0.0001	1.27
BB_0500	Ribosomal prot S13	1.25±0.11	0.0001	1.25
BB_0612	Clp protease subunit X	1.25±0.32	0.0247	1.25
BB_K45	Immunogenic prot P37, putative	1.25±0.34	0.0335	1.25
BB_A21	Predicted protein	1.24±0.1	0.0001	1.24
BB_0416	Pheromone shutdown prot	1.23±0.2	0.0028	1.23
BB_0190	Translation initiat factor IF-3	1.22±0.2	0.0041	1.22
BB_0067	Peptidase putative	1.21±0.21	0.0078	1.21
BB_0215	PstS	1.2±0.19	0.0047	1.2

Mean values and standard deviations were determined using 12 input results obtained from iTRAQ quantification values. P values were calculated based upon the 12 input results using a two-sided, one sample t test with comparison against a theoretical value of 1.0. Colored cells contain proteins whose expression is reported to be regulated only by HrpA. The complete list of proteins identified in the iTRAQ experiment is given in **Table S2**
